# Cross-feeding niches among commensal leaf bacteria are shaped by the interaction of strain-level diversity and resource availability

**DOI:** 10.1038/s41396-022-01271-2

**Published:** 2022-06-29

**Authors:** Mariana Murillo-Roos, Hafiz Syed M. Abdullah, Mossaab Debbar, Nico Ueberschaar, Matthew T. Agler

**Affiliations:** 1 Plant Microbiosis Lab, Department of Microbiology, Friedrich Schiller University Jena, Jena, Germany; 2 Mass Spectrometry Platform, Friedrich Schiller University Jena, Jena, Germany

**Keywords:** Microbiome, Plant ecology, Biodiversity, Microbial ecology, Bacterial genomics

## Abstract

Leaf microbiomes play crucial roles in plant health, making it important to understand the origins and functional relevance of their diversity. High strain-level leaf bacterial genetic diversity is known to be relevant for interactions with hosts, but little is known about its relevance for interactions with the multitude of diverse co-colonizing microorganisms. In leaves, nutrients like amino acids are major regulators of microbial growth and activity. Using metabolomics of leaf apoplast fluid, we found that different species of the plant genus *Flaveria* considerably differ in the concentrations of high-cost amino acids. We investigated how these differences affect bacterial community diversity and assembly by enriching leaf bacteria in vitro with only sucrose or sucrose + amino acids as possible carbon sources. Enrichments from *F. robusta* were dominated by *Pantoea* sp. and *Pseudomonas* sp., regardless of carbon source. The latter was unable to grow on sucrose alone but persisted in the sucrose-only enrichment thanks to exchange of diverse metabolites from *Pantoea* sp. Individual *Pseudomonas* strains in the enrichments had high genetic similarity but still displayed clear niche partitioning, enabling distinct strains to cross-feed in parallel. *Pantoea* strains were also closely related, but individuals enriched from *F. trinervia* fed *Pseudomonas* more poorly than those from *F. robusta*. This can be explained in part by the plant environment, since some cross-feeding interactions were selected for, when experimentally evolved in a poor (sucrose-only) environment but selected against in a rich (sucrose + amino acids) one. Together, our work shows that leaf bacterial diversity is functionally relevant in cross-feeding interactions and strongly suggests that the leaf resource environment can shape these interactions and thereby indirectly drive bacterial diversity.

## Introduction

In nature, plants are colonized by diverse communities of microorganisms. A balanced microbial community can work as a barrier against biotic and abiotic stress, helping to sustain plant health. On the other hand, failing to establish a normal microbiota can have devasting effects [1]. In-depth descriptive studies have shown that the composition of the plant microbiome depends on many factors [2–4], but a significant part of these differences can be attributed to networks of interactions among microorganisms [5, 6]. Besides shaping the microbiome, these interactions play critical roles in maintaining their stability, for example to invaders [6, 7] and could help explain the astounding genetic diversity found among leaf bacteria, even in single plant populations [8]. Given the importance of these interactions, increasing the understanding of how they arise and how diversity influences them, may help develop better strategies to increase plant resilience.

Microbial taxonomic diversity in plant tissues generally follows a gradient from high in roots to lower in leaves, with relatively few taxa that further colonize the leaf apoplastic space as endophytes [1, 9, 10]. This limitation is due to strong constraints on microbial life in the apoplast, including the tight regulation of nutrient resources imposed by plants, presumably in part to limit microbial growth [11–13]. Bacterial survival requires at least basic nutrients like carbon and nitrogen. Both sucrose, the primary sugar produced in leaves and amino acids are found in the apoplast [14, 15] and can play important roles in bacterial nutrition and virulence [16–18]. However, their availability is unstable and varies both between plant species and within plant species, for example due to diurnal fluctuations [19].

The lack of resources in plant apoplasts is evident when it is considered that an apparently fundamental trait of bacterial leaf pathogens is the ability to mobilize resources with the help of batteries of secreted effector proteins [20, 21]. Non-pathogenic strains (i.e., commensals), however, have fewer adaptations to manipulate plant nutrient availability and therefore are probably more directly reliant on scavenging nutrients [22]. For example, commensal *Burkholderia* and *Agrobacterium* express diverse transporters for ribose, xylose, arabinose and urea upon injection into *Arabidopsis thaliana* leaves [12]. For colonizers like these, metabolic interactions with co-colonizers are likely to play important roles helping them gain nutrition. Specifically, resource limitation increases the likelihood that cooperative or even mutualistic interactions arise, including metabolic cross-feeding [23–25]. Such cooperative interactions can also be beneficial by making communities more resilient to fluctuating nutrient availability [26]. Although it is speculated that microbe-microbe interactions in leaves involves nutrient exchange, it is not yet clear how widespread this is and whether cooperation may impact the diversity and establishment of the leaf community [27].

Here, we investigated how the apoplast nutrient environment may influence the arisal of metabolic interactions among bacteria. As a model system, we compared plant species in the genus *Flaveria* that use different photosynthesis strategies (C3: *F. robusta*, C4: *F. trinervia*). Although they are closely related, the evolution of C4 photosynthesis has had direct and indirect effects on traits like leaf metabolism [28, 29] and leaf structure [30]. Thus, we hypothesized that these differences would be likely to affect the arisal of metabolic interactions. To address this question, we used an in vitro community enrichment approach and dissected inter-bacterial interactions at the strain level using metabolomics, genomics and molecular tools. Diverse leaf bacteria have the potential to cross-feed and we found that cross-feeding potential correlated with a more nutrient-poor apoplast environment where survival of individuals is limited. Additionally, we found that metabolic interactions sustain taxonomic diversity across vastly different nutrient regimes and that partitioning of cross-feeding niches can sustain strain-level genetic diversity. Thus, our results suggest that metabolic interactions help bacteria cope with nutrient limitation in host plants and that leaf traits have the potential to shape leaf microbiomes.

## Methods

### Recovery and metabolomic analysis of leaf apoplast fluid from lab plants

*Flaveria robusta, F. linearis* and *F. trinervia* plants were generated from cuttings and grown under controlled conditions at an average day/night temperature of 25 °C/22 °C and a photoperiod of 16 h. Leaf samples were collected over the course of two years: *F. linearis* was sampled in March 2020 and July 2020; *F. robusta* was sampled in March 2020, June 2020 and April 2021 and *F. trinervia* was sampled in March 2020. Each time 2 samples were taken from 2–4 plants of each species. Apoplast fluid was extracted from fully developed leaves by infiltrating them with sodium phosphate (100 mM, pH 6.5) under vacuum in a syringe and recovering it by centrifugation, similar to Gentzel et al. [31]. An infiltration ratio, used later to correct the metabolite peak areas for the dilution that occurred during infiltration, was calculated by dividing the mass of buffer that went into the leaf over the initial leaf weight (*W*_*inf*_ − *W*_*ini*_) / *W*_*ini*_. After storage at −20 °C, the samples were subjected to metabolomic profiling via untargeted UHPLC-HRMS. Full details on the apoplast recovery as well as UHPLC-HRMS parameters and data analysis can be found in the Supplementary Methods.

### In vitro enrichment and characterization of leaf microbiomes under different nutrient regimes

#### Enrichment of leaf microbiomes from Flaveria trinervia and Flaveria robusta

Cuttings from *F. robusta* and *F. trinervia* were grown in an outdoor garden for two months (Jena, Germany) to allow natural colonization by microorganisms. From each species, fully developed leaves from different plants were collected and pooled together into single samples. Leaves were weighed and washed three times in sterile water to remove dirt and insects. Leaf microbial extracts were prepared by macerating the washed leaves in 1 X PBS + 0.02% Silwet and adding 20% glycerol before storing at −80 °C. About 1000 CFUs (estimated by plating) were pre-cultured in M9 broth with trace elements, vitamins, 11 mM sucrose, 0.2% w/v casamino acids (Difco), 200 mM NH_4_Cl and 200 µg/mL of cycloheximide to limit eukaryotic growth. After washing cells and standardizing to an OD_600nm_ of 0.3, the pre-culture was used to inoculate three replicates of the two enrichment M9 media supplemented with NH_4_Cl (33 mM) and either no casamino acids (S-CA) or 0.2% m/v casamino acids (S + CA). For each 48-h passage of the enrichment, the OD_600nm_ was recorded and 5 µL of homogenized enrichment were transferred to a new plate with fresh media. At the last passage, cells were collected for DNA extraction and to prepare glycerol stocks stored at −80 °C. Details of the entire procedure can be found in the Supplementary Methods.

#### Characterization of bacteria in original leaf extracts and in enrichments

To isolate and identify bacteria in the communities, 25 isolates were recovered from the initial leaf extract glycerol stocks and from the glycerol stocks from the twelfth enrichment passage of each condition (150 total – the number of strains were chosen based on feasibility to handle and characterize them). Isolates enriched from *F. robusta* are named FrLE, Fr-CA or Fr+CA (from leaf extracts or from enrichments without or with casamino acids, respectively) and similarly from *F. trinervia*, FtLE, Ft-CA or Ft+CA. All isolates were identified by Sanger sequencing of the 16 S rRNA gene. Additionally, bacterial communities in the twelfth enrichment passage were characterized by 16 S rRNA gene amplicon sequencing of the V3-V4 region similar to the two-step approach outlined in Mayer et al. [32] and explained in detail in the Supplementary Methods. Raw data was analyzed in R (version 4.0.4, [33]) using the package dada2 (version 1.18.0: [34] and ordination analyses and figures were created with *phyloseq* (version 1.34.0; [35]) and *vegan* (version 2.5-7; [36]). Further details are given in the Supplementary Methods.

Whole genome sequencing was performed on three *Pseudomonas* sp. (hereafter *Pseudomonas*) isolates (Fr-CA_5, Fr+CA_3 and Fr+CA_18) and four *Pantoea* sp. (hereafter *Pantoea*) isolates (Fr-CA_6, Fr+CA_20, Ft-CA_14 and Ft+CA_17). For this, DNA was extracted, purified and sent to Microbial Genome Sequencing Center (Pittsburgh, USA) for sequencing on the NextSeq 2000 (Illumina) platform at a depth of 300 MBp (~50x for *Pseudomonas* strains and ~60x for *Pantoea* strains). The genomes were each assembled using SPADES (3.14.1) and average nucleotide identity (ANI) between the different isolates of each genera was calculated in Kbase [37]. Sequence variants (SNPs and indels) compared to the *Pseudomonas siliginis* D26 reference genome (assembly accession GCF_001605965.1) were called by mapping the raw sequencing reads from each *P. siliginis* strain using SNIPPY (version 4.6.0). Further details are given in the Supplementary Methods.

### Assessment of the isolates carbon preferences and cross-feeding potential

#### Production of spent media and evaluation of cross-feeding interactions

After testing all isolates for their growth patterns in the media they were enriched in, for potential auxotrophies and sucrose utilization in presence of other nutrients (see Supplementary Methods for full details on evaluation of the isolates carbon preference) we concluded *Pseudomonas* strains must have cross-fed from *Pantoea* and this was investigated in-depth. *Pantoea* isolates were grown in S-CA (sucrose-only) media and after 48 h, the supernatant was collected by centrifugation and profiled by UHPLC-HRMS. *Pseudomonas* strains were first precultured in R2A broth and then inoculated in the *Pantoea* spent media. Growth (OD_600nm_) and metabolite consumption were recorded after 48 h. The same procedure was followed to assess the cross-feeding potential of several leaf-extract isolates (FrLE and FtLE) towards *Pseudomonas*.

### Competition between *Pseudomonas* strains while cross-feeding

To evaluate whether fast growing *Pseudomonas* would outcompete slow-growing strains while cross-feeding from *Pantoea*, we tagged the isolates *Pseudomonas* Fr-CA_5 (fast grower) and *Pseudomonas* Fr+CA_3 (slow grower) with the fluorophores mTagBFP2 and mOrange2, respectively, making them clearly distinguishable for colony counting. We used the delivery plasmid systems pMRE-Tn7-140 and pMRE-Tn7-144 developed by Schlechter et al. [38] (see Supplementary Methods for details). Next, we set three different experiments: in the first one, the tagged strains were combined individually with *Pantoea* Fr-CA_6 or together in a full mix (both *Pseudomonas* strains and *Pantoea*) and inoculated in black 96-well plates containing S-CA media. The fast-growing *Pseudomonas* isolate was added at half the density of the slow-growing isolate. The plate was incubated in a BioLector I (m2p-labs Beasweiler, Germany) at 500 rpm, 30 °C with humidity control. The controls (each *Pseudomonas* alone with *Pantoea* and *Pantoea* alone) were passaged every 24 h eight times, while the full community was also passaged four times every 48 h. After each passage, samples were taken for CFU counts. Growth of *Pseudomonas* Fr+CA_3 was monitored by normalizing the red filter channel signal to the biomass signal (more details are given in the Supplementary Methods).

In the second experiment, the two tagged *Pseudomonas* strains were grown either in monoculture or in co-culture in S + CA over four 24-h passages. This experiment was carried out in clear 96-well plates and incubated in an orbital shaker at 28 °C and 220 rpm. The OD_600nm_ and the fluorescence of the mTagBFP2 and mOrange2 were read in a Tecan Plate Reader at the time of passaging (more details in Supplementary Methods).

For the third experiment, the fast growing strains (*Pseudomonas* Fr-CA_5:mTagBFP2 and *Pseudomonas* Fr+CA_18) and the slow growing strains (*Pseudomonas* Fr+CA_2 and *Pseudomonas* Fr+CA_3:mOrange2) were combined in all possible pairs and passaged six times on *Pantoea* Fr-CA_6 spent media. Each strain was also inoculated in monoculture. The incubation conditions, as well as the monitoring parameters for OD_600nm_ and fluorescence, were the same as in the previous experiment. The used media from the 1st passage was centrifuged (5000 rpm for 5 min) and 40 µL of the supernatant were collected for UHPLC-HRMS analysis. The last passage was plated out in LB agar to count CFUs of each strain.

### Evaluating link of cross-feeding efficiency and colonization to host species

#### *In-planta* testing of *Pseudomonas* colonization

We tested the ability of two *Pseudomonas* strains: Fr-CA_5 and Fr+CA_3 to persist in leaves of *F. robusta* or *F. trinervia*. For this we inoculated three-week old cuttings grown in potting soil with a washed cell suspension of either *Pseudomonas* alone or in combination with *Pantoea* (OD_600nm_ 0.002 of each). The plants were kept in a growth chamber. After 10 days, the leaves which had been inoculated were harvested, surface sterilized first with 2% bleach + 0.02% Triton, followed by 70% ethanol to remove surface bacteria and then washed three times with sterile water. They were then processed to obtain CFU counts/g of leaf. The inoculation was repeated in two fully independent experiments for each species. See Supplementary Methods for full details.

#### Assessing cross-feeding potential of *Pantoea* isolates

Following the same procedure as before, *Pantoea* isolates from all enrichments (Fr + CA, Ft -CA and Ft +CA) were tested for their potential to cross-feed *Pseudomonas*. Samples of the supernatant were taken after 48 h and subjected to UHPLC-HRMS. To test for inhibitory effects, the spent media of the isolates *Pantoea* Ft-CA_14 and *Pantoea* Ft+CA_17 were diluted 1:2 with spent media of *Pantoea* Fr-CA_6 before growth of *Pseudomonas*. Full details of these experiments are specified in the Supplementary Methods.

### *Pantoea* and *Pseudomonas* experimental adaptation

To address whether the nutrient environment affects the cross-feeding interactions, we passaged the two tagged *Pseudomonas* (Fr-CA_5:mBFP2 and Fr+CA_3:mOrange2) together with either *Pantoea* Fr-CA_6 or *Pantoea* Fr+CA_20 on the two different media: S-CA or S + CA. The precultures were prepared and diluted as before, but in this case, 10 µL of the *Pantoea* suspension (OD_600nm_ = 0.2) were mixed with 5 µL of each *Pseudomonas* strain (OD_600nm_ = 0.2) and inoculated into 180 µL of the corresponding media. Each community had four replicates. The communities were passaged every 24 h for 25 days. At passage 22, the communities that had been growing in S-CA were transferred to S + CA and vice versa. The OD_600nm_ and fluorescence were measured as before (See also Supplementary Methods).

## Results

### Leaf apoplasts have plant species-specific availability of potential nutrient resources

**Fig. 1:**
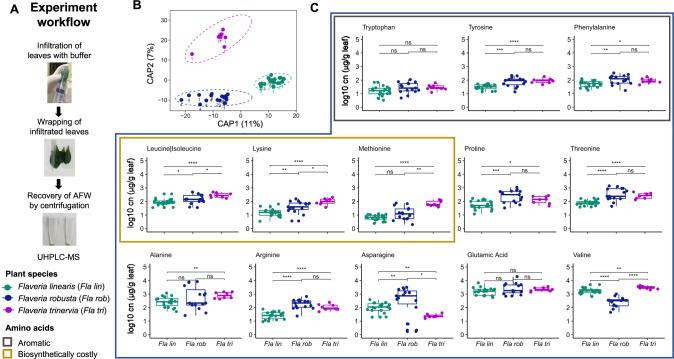
*Flaveria* species have distinct metabolic composition of the apoplast fluid of leaves. **A** Infiltration of leaves and recovery of apoplast fluid wash. **B** Principle coordinate analysis on Euclidean distances, constrained by plant species and based on all UHPLC-HRMS peaks (area > 1.0E4) in untargeted metabolomics of the extracted leaf apoplast. Points represent replicate sampling at a given time point, ellipses show 95% confidence intervals, numbers indicate the sampling event: *N* = 20 *F. linearis*, *N* = 18 *F. robusta*, *N* = 8 *F. trinervia*. A generalized logarithm transformation (Parsons et al. 2007) was applied to the data. **C** Amino acids concentrations in the apoplast of *F. trinervia, F. robusta* and *F. linearis*. Concentrations were calculated based on an internal standard. Significance values are based on Kruskal Wallis test comparing concentrations between species (ns: *p* value > 0.05, *p < = 0.05, **p < = 0.01, ***p < = 0.001).

To get a first idea of the nutrient environment that leaf colonizing bacteria are exposed to upon entering the apoplast, we extracted apoplastic fluid from three species of *Flaveria* that each use distinct photosynthesis pathways (Fig. 1A). *F. robusta* utilizes C3 photosynthesis, *F. trinervia* utilizes C4 photosynthesis and *F. linearis* uses an “intermediate” photosynthesis strategy. Compounds were identified as distinct peaks in the untargeted UHPLC-HRMS profiles. Constrained analysis of principal coordinates based on all detected compounds showed an overall clear separation of the plant species (Fig. 1B, PERMANOVA *p* value = 9.999e-05). Using a targeted approach in parallel, we could quantify amino acids in the extracted apoplastic fluid. Among all detected amino acids, there were significant differences between plant species (Kruskal Wallis *p* value < 0.05) in all except Glu and Trp. Among the seven amino acids with highest metabolic costs (56) the aromatics (Tyr, Trp and Phe) were significantly more abundant in *F. trinervia* than in *F. linearis* and tended to be higher in *F. robusta* (Fig. 1C). Three of the other four could also be detected (Met, Ile, Lys, not His) and all were significantly higher abundance in *F. trinervia* than the other two species (Fig. 1C). Thus, the nutrient landscape for potential leaf apoplast colonizers differs between plant species, with some like *F. trinervia* potentially offering higher amino acid availability.

### Nutrient and taxonomic diversity shapes microbiome function in *Flaveria* leaf enrichments

**Fig. 2:**
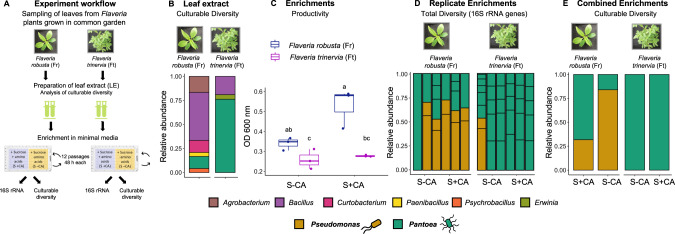
Bacterial community enrichments from *F. robusta* leaves show higher productivity and more functional responsiveness to nutrient diversity than *F. trinervia*. **A** Enrichment of leaf bacterial communities in two different minimal media. **B** Culturable diversity in the leaf extracts used for enrichment from *Flaveria robusta* and *F. trinervia*, based on 16 S rRNA Sanger sequencing; *n* = 25 isolates. **C** Productivity (final optical density at OD_600nm_) of the enrichments from each species. Different letters indicate significantly different values (Kruskal Wallis test with FDR adjustment < 0.05, *N* = 3) (**D**) Diversity obtained in each enrichment replicate after 12 passages based on amplicon sequencing of 16 S rRNA gene. **E** Culturable diversity obtained in each enrichment after 12 passages based on Sanger sequencing of 16 S rRNA gene (Three replicates were combined for isolation; *n* = 25 isolates). The taxonomy color legend corresponds to panels (**B**, **D** and **E**).

We tested whether and how the nutrient landscape can influence assembly of leaf bacterial communities (Fig. 2A). We enriched bacteria derived from leaves of *F. trinervia* and *F. robusta* (Fig. 2B) in a base minimal media with sucrose as the only carbon source and either no amino acids (S-CA) or addition of casamino acids (S + CA) at low levels similar to those previously reported in the apoplast [39]. The final OD_600nm_ was used as a measure of productivity (function) of the enrichments (Fig. 2C). In the S-CA condition where sucrose was the only carbon source, the *F. robusta* enrichment was slightly more productive than the *F. trinervia* enrichment. Additionally, enrichments from *F. robusta* tended to increase their productivity when the potential carbon and nitrogen sources were richer (S + CA), but those from *F. trinervia* did not.

To have a broader picture of their taxonomic composition, we conducted amplicon sequencing on the enriched communities (Fig. 2D). Despite diverse bacteria in the original leaves (Fig. 2D), enrichments selected very few taxa, which was not affected by additional resource availability in the form of amino acids. *F. trinervia* enrichments were fully dominated by *Pantoea* sp. (Fig. 2D). *F. robusta* enrichments also contained a high prevalence of *Pantoea*, but in combination with *Pseudomonas* (Fig. 2D). To investigate why the productivity differed between the enrichments, we collected 25 random bacterial isolates from the *F. robusta* and *F. trinervia* S-CA and S + CA communities (isolates designated as Fr-CA_X, Fr+CA_X, Ft-CA_X or Ft+CA_X, where X is a unique isolate number), identified them taxonomically (Fig. 2E) and tested the growth of each in S-CA and S + CA (Supplementary Table 2). The isolates included multiple individuals of both *Pseudomonas* and *Pantoea*, the only genera that were found using 16 S data. In the S-CA community, the fraction of *Pantoea* was higher than in S + CA (67% vs. 16%, respectively, Fig. 2E). All tested *Pantoea* isolates could grow on sucrose or amino acids as a sole carbon source (Supplementary Table 2). The fact that productivity did not increase in *F. trinervia* enrichments with casamino acid addition suggests that another resource must have limited additional growth. All tested *Pseudomonas* isolates could use amino acids as a sole carbon source (Supplementary Table 2). Thus, the increased productivity in the *F. robusta* S + CA enrichment suggests that they could make use of the additional resources. *Pseudomonas* isolates did not use sucrose as a sole carbon source even though they were present in the S-CA (sucrose-only) enrichment (Supplementary Table 2). Therefore, *Pseudomonas* in this enrichment may have been somehow dependent on *Pantoea*, which could explain the higher productivity in *F. robusta* enrichments compared to *F. trinervia* when sucrose was the only carbon source.

### Cross-feeding on diverse metabolites sustains *Pseudomonas* in the absence of a primary carbon source

We then asked how *Pseudomonas* isolates survived in the S-CA enrichment if they could not utilize sucrose directly. One possibility is they were auxotrophs and could only grow when amino acids were available in the environment (either in S + CA or provided from *Pantoea* in S-CA). However, all tested *Pseudomonas* isolates could grow on glucose without amino acid supplementation (Supplementary Table 2), suggesting that they were not strict auxotrophs.

**Fig. 3:**
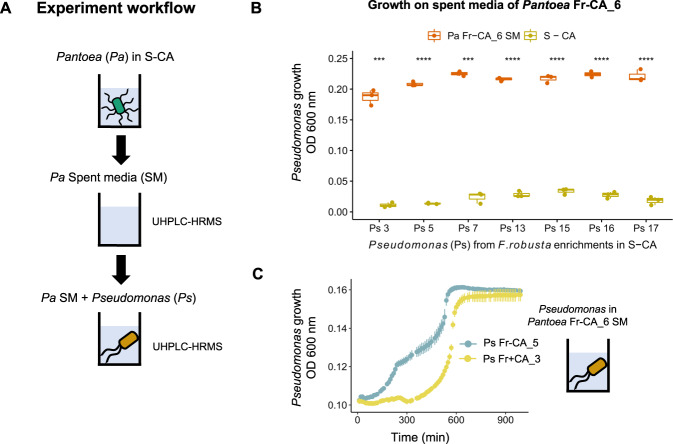
*Pseudomonas* isolates could persist in the *F. robusta* -CA enrichment by cross-feeding with *Pantoea* isolates. **A** Procedure to obtain *Pantoea* (*Pa*) spent media and evaluate its potential to support *Pseudomonas* (*Ps*) growth. **B** Growth of *Ps* isolates from the Fr-CA enrichment on Pa Fr-CA_6 spent medium. Significance values are based on a *t*-test comparing growth in S-CA and in *Pa* Fr-CA_6 spent medium (ns: *p* value > 0.05, *p < = 0.05, **p < = 0.01, ***p < = 0.001, *N* = 3). **C** Growth curves of two *Pseudomonas* isolates (*Ps* Fr-CA_5 and *Ps* Fr+CA_3) over the course of 24 h on *Pa* Fr-CA_6 spent medium. Each data point shows the mean of three replicates and the corresponding standard error. OD_600nm_ refers to optical density measured at 600 nm.

We tested whether *Pantoea* produced other metabolic by-products that *Pseudomonas* could grow on (Fig. 3A). Indeed, all *Pseudomonas* isolates from the sucrose-only enrichment (*Pseudomonas* Fr-CA) could grow on spent media of a *Pantoea* isolated from the same enrichment (*Pantoea* Fr−CA_6, Fig. 3B). To determine what *Pseudomonas* consumed, we performed untargeted metabolomics on the spent media before and after growth of three *Pseudomonas* isolates and considered metabolite peaks to be consumed if their area was strongly reduced (log2FC < −2, FDR < 0.05). With this strict cutoff, we detected uptake of between 23–25 metabolites by each strain, including 2-hydroxybutyric acid, hypoxanthine, spermidine and, in one of the isolates, alanine (Supplementary Table 3). This indicates a complex metabolic dependency of *Pseudomonas* on *Pantoea* in the sucrose-only enrichments. We did detect trace levels of glucose and/or fructose in the *Pantoea* spent medium (hexoses are indistinguishable with this method), but they were not significantly taken up (log2FC = −0.85, *p* value = 0.36 on average, not shown).

Several isolates from the original leaf extracts (FrLE and FtLE) could also feed *Pseudomonas*. The growth on *Bacillus* FrLE_12 spent medium, for example, was especially high, even compared to growth on *Pantoea* spent medium (Supplementary Fig. 1). Metabolomic analyses suggested that *Pseudomonas* may utilize a diverse array of metabolites from this isolate (log2FC < −2, FDR < 0.05) with little or no overlap with compounds utilized from *Pantoea* spent media (Supplementary Table 4). None of the taxa with potential to cross-feed *Pseudomonas* were found in the enrichments, probably due to their lower growth rate in sucrose, when compared to *Pantoea* (Supplementary Fig. 1). Overall, these results suggest that when competition for sucrose dominates dynamics, *Pseudomonas* will be limited in cross-feeding partners but otherwise may be able to persist by feeding on exudates of diverse leaf bacteria.

### Diverse plant-derived *Pseudomonas* strains cross-feed in parallel from *Pantoea*

In the S-CA enrichment, *Pseudomonas* survived exclusively by cross-feeding on diverse resources from *Pantoea* exudates, but in the S + CA enrichment, it would have been able to either cross-feed or utilize amino acids or both. Therefore, we hypothesized that multiple *Pseudomonas* strains may exist to optimally utilize this niche diversity. Indeed, we observed that two *Pseudomonas* isolates displayed distinct growth phenotypes on *Pantoea* spent medium. *Pseudomonas* Fr-CA_5 grew earlier and reached maximum OD_600nm_ faster compared to *Pseudomonas* Fr+CA_3 (Fig. 3C and Supplementary Fig. 2a). A correlated phenotype was observed in R2A medium, where only the slower cross-feeder switched to more rapid growth when supplemented with vitamins that were present in the enrichment medium (Supplementary Fig. 2b). We predicted that in the S-CA enrichment where cross-feeding was required, faster cross-feeders would outcompete slower ones to dominate the mix, while the more diverse nutrient conditions in the S + CA enrichment would result in a more balanced mix. However, when we checked the phenotype across all *Pseudomonas* isolates, we found that the strains were in similar ratios in both enrichments (Supplementary Fig. 2c, X^2^ *p* = 1).

**Fig. 4:**
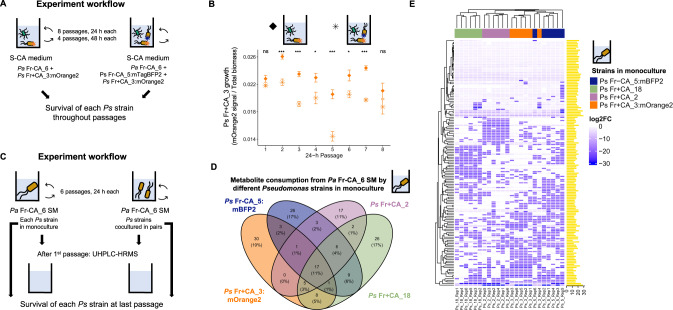
Different *Pseudomonas* (*Ps*) isolates had specialized niches when feeding from *Pantoea* (*Pa*) Fr-CA_6. **A** Passaging of tagged *Ps* strains along with *Pa* Fr-CA_6 on S-CA medium. **B** Signal of *Ps* Fr+CA_3:mOrange2 fluorescence normalized by the biomass signal in either co-culture with *Pa* Fr-CA_6 or in the full community with *Pa* Fr-CA_6 and *Ps* Fr-CA_5:mTagBFP2. **C** Each *Pseudomonas* strain was passaged either alone or in pairs with each other on *Pa* Fr-CA_6 spent medium. **D**, **E** Taken up metabolites from *Pa* Fr-CA_6 spent media by each *Ps* strain when grown in monoculture. Only metabolites with significant uptake are shown (log2 FC < −2, FDR < 0.05). In the heatmap, each column represents a different repetition (*N* = 6 for each strain). The strains and peaks have been clustered by Euclidean distance. The yellow bars on the right indicate the area (log10) of the peak in the spent media of *Pantoea* Fr-CA_6 before growth of the *Ps*. The information of each peak ionization mode, retention time, m/z value and putative annotation) is given in Supplementary Table 6. The order of the peaks shown in the heatmap matches exactly the order of the peaks in the Table.

To test whether faster and slower-growing strains could simultaneously cross-feed from *Pantoea*, we labeled the slow and fast cross-feeder (*Pseudomonas* Fr+CA_3 and *Pseudomonas* Fr-CA_5) with fluorescent tags, which did not alter their growth patterns on *Pantoea* spent media (Supplementary Fig. 3a). We then combined the strains and grew them directly with *Pantoea* Fr-CA_6 in sucrose-only media (Fig. 4A). Although the fast cross-feeder was inoculated with only half the cells as the slow cross-feeder (see Supplementary Methods), they were approximately equivalent within 48 h (Supplementary Fig. 3B). However, neither strain overtook the other after four 48 h or eight 24 h passages (Supplementary Fig. 3b, c, raw signals provided in Supplementary Fig. 4). In the 24 h passages, the slow grower did reach higher levels with *Pantoea* alone than it did together with the fast grower (Fig. 4B), consistent with a hypothesis of partially overlapping niches. Total biomass was higher when *Pantoea* was with *Pseudomonas* than alone, similar to productivity observations in the original enrichments (Supplementary Fig. 4). Interestingly, the strains could also persist together in the more complex S + CA media without *Pantoea* (Supplementary Fig. 5).

Differential niches is also supported in genetic analysis of two fast-growing and one slow-growing strain. All were identified as *P. siliginis* and shared 99.98 to 99.99% ANI (Supplementary Table 5). Aligned to the closest reference *Pseudomonas* genome, they shared about 97,000 sequence variants with only a few hundred unique to any one genome (Supplementary Fig. 6). Among unique and potentially disruptive variants in each strain, many were in genes likely to influence nutrient acquisition, including porins and carboxylate, sugar and peptide transport proteins (Supplementary Fig. 6 and Supplementary Methods). Thus, we reasoned that the strains must have unique nutrient niches whereby fast-growing strains can dominate only some resources, reducing the growth of the slow-growers when they are together, but the slow-growers can persist because they also use unique resources.

### Niche differentiation among distinct *Pseudomonas* strains maintains diversity during cross-feeding

To test whether and how *Pseudomonas* strains exploit complementary niches, we cultured the two labeled *Pseudomonas* (Fr-CA_5 and Fr+CA_3) and two additional fast and slow growing isolates (*Pseudomonas* Fr+CA_18 and *Pseudomonas* Fr+CA_2, respectively) individually and in all possible pairs in *Pantoea* spent media (Fig. 4C) and evaluated metabolite uptake (niches) and growth patterns. The pattern of metabolite uptake by the strains growing alone was consistent with partially overlapping niches. Several significantly taken up metabolite peaks were shared by all four isolates (common niches) and each also significantly depleted unique sets of metabolite peaks (unique niches) (Fig. 4D, E and Supplementary Table 6). When grown in pairs (Supplementary Figs. 7–12), most of the common niches and some of the unique niches were maintained, further supporting partially overlapping niches. However, new compounds were also taken up, suggesting some cooperative niche exploration. Similar to previous results (Supplementary Table 3), the annotated peaks matched to purines and pyrimidines and to amino acids Trp and Ile/Leu in some combinations (Supplementary Table 6). Over 6 24-hr growth cycles, growth of individual tagged strains was reduced in co-cultures compared to monocultures (based on both fluorescence signal and CFU counts, Supplementary Figs. 13 and 14) but both strains in co-cultures successfully persisted, further supporting a lack of competitive exclusion. Notably, *Pseudomonas* Fr+CA_2 alone or in co-culture had lower total growth (CFUs) than other combinations (Supplementary Fig. 14). This was apparently not because of inhibition, since growth of other strains with Fr+CA_2 spent media was rather promoted (Supplementary Fig. 15). A likely explanation is that Fr+CA_2 inefficiently used some limiting resource thereby reducing growth of itself and co-colonizers. Regardless, this further highlights the extent of *Pseudomonas* commensal strain diversity.

### Cross-feeding is selected for in a leaf nutrient-dependent manner

Two additional lines of evidence suggested that the plant nutrient landscape could actively select on cross-feeding interactions. First, *F. robusta* and *F. trinervia* leaves were inoculated with a slow- or a fast-growing *Pseudomonas* with or without a partner *Pantoea* strain and endophytic growth was measured (Supplementary Fig. 16a). Alone, each of the two *Pseudomonas* strains successfully colonized *F. robusta* leaves only occasionally, but consistently colonized *F. trinervia* leaves at high levels (2-sided *t*-test, *p* value = 0.06 and 0.05, respectively). We did not observe differences in *Pseudomonas* colonization when it was inoculated together with *Pantoea*, but this is likely due to very variable colonization success (Supplementary Fig. 16a). The inability of a commensal to colonize the relatively less nutrient-rich *F. robusta* alone could increase possible dependence on partners. Second, we found among four characterized *Pantoea* isolates (one from each enrichment) a gradient in their ability to feed *Pseudomonas* corresponding to the richness of the origin plant and the enrichment condition. *Pantoea* from *F. robusta* fed *Pseudomonas* best (Fr-CA better than Fr+CA) while *Pantoea* from *F. trinervia* supported *Pseudomonas* hardly or not at all (Supplementary Fig. 16b). Additional analyses suggest that differences are likely due to specific exudates, not antagonistic traits (Supplementary Note). The cross-feeding differences between the *Pantoea* strains do not correlate to their genetic relatedness: The two *Pantoea* Fr strains that best support *Pseudomonas* growth shared only ~81% ANI, but *Pantoea* Fr-CA_6 and the poor cross-feeding *Pantoea* Ft isolates share ~98.6% ANI (Supplementary Table 7), consistent with good cross-feeding emerging as a convergent trait in *Pantoea* in *F. robusta*.

**Fig. 5:**
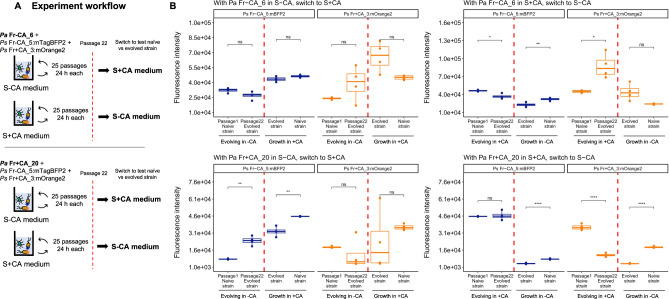
Cross-feeding interactions between *Pantoea* and *Pseudomonas* followed distinct paths during evolution in two nutrient environments. **A** Passaging of the two tagged *Pseudomonas* (*Ps*) strains with either *Pantoea* (*Pa*) Fr-CA_6 or *Pa* Fr+CA_20 on S-CA or S + CA medium. The red vertical line marks passage 22, when the communities were switched to the opposite media to evaluate the effects of their adaptation. **B** Fluorescence signals of *Ps*Fr-CA_5:mTagBFP2 and *Ps* Fr+CA_3:mOrange2 detected in the communities. Passage 1 naïve strain: Fluorescence intensity of the strain at the beginning of the experimental adaptation, Passage 22 evolved strain: Fluorescence intensity after 22 passages in the experimental adaptation right before switching media. Comparison of Passage 1 naïve and Passage 22 evolved, show changes in strain fitness over the experimental adaptation. Evolved strain after the switch of media: Fluorescence intensity after the evolved community was switched to the other nutrient condition and allowed to equilibrate for 4 passages. Naïve strain: Fluorescence intensity of the naïve strain in the media that the evolved strain was switched to. Comparison of evolved strain after the switch and the naïve strain shows how adaptation in one nutrient condition affected fitness in the other one. Significance values are based on *t*-tests comparing the fluorescence intensity between Passage 1 and Passage 22 (evolving media) and the evolved and naïve strains in the switch media. ns: *p* value > 0.05, *p < = 0.05, **p < = 0.01, ***p < = 0.001, *N* = 4.

Next, we tested the hypothesis that the cross-feeding interaction can be selected on by the nutrient environment by evolving two communities across 25 24-hr passages (the tagged *P. siliginis* Fr-CA:5mBFP2 and *P. siliginis* Fr+CA_3:mOrange2 both together with either Pantoea Fr-CA_6 or Pantoea Fr+CA_20) in two contrasting nutrient environments (S-CA where cross feeding is required or S + CA where it is not necessarily needed) (Fig. 5). At the 22nd passage, the evolved communities were switched to the opposite environment for 4 cycles. Thus, we obtained two outputs: (1) How growth of the *Pseudomonas* strains with *Pantoea* changed (based on fluorescence over the 25 cycles) and (2) How changes affected growth in the other environment (based on fluorescence after switching). (Fig. 5 and see additional details in Supplementary Notes and raw growth data in Supplementary Fig. 19). *Pseudomonas* growth could change due to adaptation of either *Pantoea* or *Pseudomonas* or both, so here we refer just to interaction fitness.

Although each *Pantoea* was always simultaneously evolved together with two *Pseudomonas* strains, each interaction clearly followed distinct paths (Fig. 5B). The *Pseudomonas* Fr-CA:5mBFP2 interaction generally fit our hypothesis for how environment should select on cross-feeding. When evolved in the -CA environment, the cross-feeding interaction exhibited either no change or increased (with *Pantoea* Fr-CA_6 and Fr+CA_20, respectively) and this came at a cost to fitness in the +CA environment. When evolved in the richer +CA environment, fitness did not change or decreased only slightly (with *Pantoea* Fr-CA_6) over 25 cycles, but the cross-feeding interaction significantly degraded (evidenced by lower growth of the evolved strain after the switch compared to the naive strain). The *Pseudomonas* Fr+CA_3:mOrange2 interaction, on the other hand, evolved in a way that was very dependent on the *Pantoea* isolate. With *Pantoea* Fr-CA_6, fitness increased over the experiment in both environments (3/4 replicates in −CA and 4/4 replicates in +CA) apparently because fitness was coupled across the environments (evolved replicates that grew better in one environment also grew better in the other environment after switching). With *Pantoea* Fr+CA_20, fitness decreased over the course of the experiment (3/4 replicates in −CA and 4/4 replicates in +CA) with evolved fitness in each environment again strongly correlated to fitness after the switch between environments. Together, these results demonstrate a more complex but consistent story of selection: Selection for cross-feeding depends on amino acid richness for some strains, while for others it is independent of richness but highly dependent on the cross-feeding partner.

## Discussion

Leaf bacteria play important roles in protecting plants from pathogens and stress via a variety of different mechanisms, so there is broad interest in understanding the factors that shape their diversity and abundance [40]. It is increasingly clear that interactions between microbial colonizers play major roles in community structuring, whereby the presence of specific microorganisms can drastically alter leaf communities [5]. However, the complications of studying microbe-microbe interactions in-vivo in leaves severely limits our understanding of the types of interactions that are important. To overcome this, we used in vitro enrichment of leaf bacteria under defined nutrient regimes and could demonstrate that leaf bacteria interact with one another via metabolic cross-feeding. This supports previous work that showed that pervasive cross-feeding is possible between leaf-derived bacteria [41] and that it can explain how a surprising diversity can subsist on single carbon sources. Our work additionally showed that cross-feeding can support bacterial taxa who have no direct utilizable carbon source and that in this case, they can survive by utilizing only secreted or leaked metabolites from diverse bacteria. We also showed that environmental conditions relevant in plants can exert selection pressures on cross-feeding interactions in bacterial strain-dependent ways.

The cross-feeders in our enrichments consistently were only *Pantoea* and *Pseudomonas*. This was surprising to us, since in previous studies, the diversity of cross-feeding taxa enriched on single carbon sources was high due to promiscuous cross-feeding interactions [41, 42] and because of our finding that multiple other bacterial taxa that we isolated from leaves could have fed *Pseudomonas*. The most likely explanation is simple resource competition. *Pantoea* grew much faster than other leaf isolates on sucrose, allowing it to become the sole feeder of *Pseudomonas*. *Pseudomonas* also grew rapidly on *Pantoea* spent medium and could have thereby outcompeted others for key resources. Our results do not necessarily mean that cross-feeding partners would be this restricted in leaves. In contrast to the well-mixed and homogeneous in vitro enrichment environment where competition would dominate dynamics, the leaf apoplast is highly compartmentalized and heterogeneous [43]. Additionally, most endophytic commensal bacteria in leaves reach only low colonization density [44]. These factors would decrease the importance of resource competition and thereby diverse taxa would be able to establish a niche consuming primary plant-derived resources and *Pseudomonas* or others could establish to take up leaked metabolites. Therefore, cross-feeding interactions in leaves can potentially arise between taxonomically much more diverse members than what we observed in enrichments.

It is important to note that our findings that bacterial diversity is relevant for metabolic interactions among very common leaf bacteria is based on a culture-dependent approach and a limited set of isolates. While such culture-dependent work is critical especially given technological barriers that limit our ability to directly observe metabolic interactions in leaves, it also means that we cannot yet fully weigh the relative importance of metabolic or other (e.g., competitive) interactions among our isolates or especially uncultivated microbes. On the other hand, culture independent techniques and modeling can help provide some insight into the relevance of such interactions. Generally, inference of inter-bacterial interactions in leaves based on culture-independent taxa correlations has suggested extensive negative interactions, but recent improvements that utilize abundance information suggests that positive interactions have probably been underestimated [44]. Similarly, experimental results suggest frequent positive interactions among co-colonizing leaf bacteria [45, 46]. This might seem unlikely, since ecological models have predicted that cooperative interactions can destabilize microbial communities and that competition should therefore benefit hosts [47]. However, the instability in these models is caused by strong species dependencies that can easily be interrupted. In contrast, cross-feeding among leaf bacteria seems to involve weak coupling and high promiscuity.

This type of cross-feeding involving promiscuous interactions could even benefit host plants by stabilizing microbiomes to invasion if cross-feeding networks are redundant enough to leave few resources for invaders [48]. Additionally, apoplast nutrients are important regulators of pathogen virulence, so full occupation of resources by cross-feeding could directly limit damage caused by them even if they successfully invade [49]. This could explain, for example, how *Pantoea* protects crops from pathogenic *P. syringae* by decreasing its virulence without eliminating it from the leaf microbiome [50]. Additionally, cross-feeding would be beneficial if it allows bacteria to establish who can, upon invasion, switch to competitive behaviors that protect plants, such as antibiotic production [51]. Therefore, more thorough investigations into the role of cross-feeding in interactions between leaf bacteria and pathogens and its general role in shaping and stabilizing co-colonizing leaf microbiota are needed, including dissection of how metabolic networks arise from host resources through the microbiome.

The three *Pseudomonas siliginis* isolates we sequenced were genetically similar with ANI of ~99.98% to 99.99% and ~97,000 shared SNPs against the most similar *P. siliginis* reference genome. While more study is needed to understand this diversity, it appears to be functionally relevant. The sequenced strains had distinct cross-feeding niches with only partially overlapping metabolite uptake profiles, making it possible to cross-feed in parallel despite different growth rates. In addition, two of the strains each showed clearly distinct patterns of adaptation when evolved with *Pantoea* in two nutrient environments. This diversity must have originated in the original leaf communities since the phenotypes appeared both in sucrose-only enrichments where cross-feeding was required and in enrichments with amino acids as additional resources. Similar levels of diversity were previously found in leaf-associated *Pseudomonas viridiflava*, where a single OTU (99% 16 S rRNA gene sequence similarity) harbored at least 82 distinct strains (99.9% genome identity), with clearly different host interaction phenotypes [8]. Commensal *Sphingomonas* bacteria were also shown to exhibit extensive diversity in the presence of secretion systems likely relevant for microbe-microbe interactions [52]. Given that diverse leaf bacteria from multiple plant species have been shown to readily engage in cross-feeding [41], our results strongly suggest that leaf bacterial genetic diversity has important functional relevance for inter-bacterial metabolic interactions in leaves and probably wherever they persist. Since nutrition plays key roles in virulence [53, 54], it also raises the intriguing question of whether interactions like cross-feeding may ultimately influence interactions with hosts.

*Pantoea* isolates also exhibited interesting and surprising functional diversity. Those from *F. robusta* enrichments, where cross-feeding occurred, fed *Pseudomonas* better than those from *F. trinervia* enrichments, probably by producing more of key metabolites. However, two “good” cross-feeders enriched from *F. robusta* were only distantly related (81% ANI), consistent with inter-species differences [55] compared to 98–99% ANI between good and bad cross-feeders. This result is consistent with the *F. robusta* environment selecting for *Pantoea* with increased metabolite secretion, benefitting cross-feeders. This agrees with *in-planta* data, where individual bacteria persisted in *F. trinervia* leaves alone at higher levels than in *F. robusta*, possibly due to relatively lower levels of key nutrients like amino acids methionine, isoleucine, lysine and valine. Except for the aromatic amino acids, methionine, isoleucine and lysine are three of the four biosynthetically highest-cost amino acids (together with histidine, [56]. The availability of these amino acids could also directly affect exchange of the diverse metabolites we observed here. For example, cross-fed purines guanine and hypoxanthine are connected to methionine via the THF cycle. Thus, differences in the apoplast metabolite landscape could alter selection for traits underlying cross-feeding. Indeed, experimental evolution of cross-feeding interactions in contrasting nutrient environments confirmed that the nutrient environment and the specific cross-feeding interaction determine the adaptive path followed by the strains.

In extreme cases, increased metabolite secretion could result from evolution of reciprocal cross-feeding between *Pantoea* and *Pseudomonas* specifically, which can in turn lead to strong co-adaptation [23]. However, this seems unlikely in leaves since conditions including compartmentalization, low bacterial density and high bacterial diversity are likely to make interactions between specific taxa transient, decreasing the likelihood that reciprocal cross-feeding will arise [57]. Thus, if more metabolite secretion is a beneficial adaptation in the *F. robusta* apoplast environment, it is more likely because *Pantoea* derives benefits from cross-feeding with diverse taxa rather than *Pseudomonas* alone.

It is important to note that our results were based on a single set of enrichments from *Flaveria* plants and therefore represent only a sliver of the interactions that probably happen in a more diverse leaf microbiome and across more diverse host plants. However, given the clear evidence that microorganisms influence one another’s colonization patterns in leaves [5, 6] and the promiscuity of cross-feeding among leaf bacteria, it seems highly unlikely that the vast genetic diversity and stable phenotypes known to exist among leaf bacteria [8] only developed in response to interactions with hosts. Whether the most important interactions shaping this diversity are cooperative metabolic interactions or some other interactions type remain to be seen and this will be an important future avenue of research. However, our results that nutrient differences that are relevant in host plants can drive divergent adaptive cross-feeding responses in closely related bacterial strains add weight to the argument that metabolic interactions may plant important roles.

The apoplast is together with the roots a critical site of host interaction with microbes. While it is not yet clear why some amino acids and other metabolites differ strongly between *Flaveria* species, one possibility is their different photosynthesis mechanisms. Evolution of C4 photosynthesis in *Flaveria* has had diverse effects. For example, higher glutathione turnover in sulfate assimilation [28] has led to higher cysteine levels in *F. trinervia* leaves. While we could not reliably quantify cysteine, it is the direct precursor to methionine, which was significantly elevated in *F. trinervia* apoplast. Thus, links between photosynthesis and apoplast metabolites are plausible. This is reminiscent of roots, where the exudate nutrient landscape is key to shaping microbial communities [58, 59] and differs between different plant species [60, 61]. Our results strongly suggest that apoplast exudates influence the arisal of microbial interactions, which in turn help shape leaf microbiomes [5]. If so, this is an exciting prospect because it could offer targets for manipulation by plant breeders.

## Supplementary information

Supplementary Information

Supplementary Table 2

Supplementary Table 3

Supplementary Table 4

Supplementary Table 6

Supplementary Table 8

Supplementary Table 11

## Data Availability

Supplementary methods, figures and tables are provided in the files Supplementary Information and Supplementary Tables 2, 3, 4, 6, 8 and 11. Scripts and data used to recreate all figures and supplementary tables are publicly available via Figshare: https://figshare.com/projects/Niche_separation_in_cross-feeding_sustains_bacterial_strain_diversity_across_nutrient_environments_and_may_increase_chances_for_survival_in_nutrient-limited_leaf_apoplasts/125920. Raw sequencing data has been made publicly available in the NCBI BioProject PRJNA778092 and raw metabolomic data is being made publicly available in MetaboLights under the study MTBLS3719.
