# Supplementary material for: Cross-feeding niches among commensal leaf bacteria are shaped by the interaction of strain-level diversity and resource availability
Source: ISME J. 2022 Jun 29;16(9):2280–9. doi: 10.1038/s41396-022-01271-2 (PMC9381498; doi:10.1038/s41396-022-01271-2)
Supplement: Supplementary file 1 — Supplementary Information [file 41396_2022_1271_MOESM1_ESM.pdf]

Supplementary Information for: Cross feeding niches among commensal leaf bacteria are shaped by the interaction of strain-level diversity and resource availability

Mariana Murillo-Roos[1], Hafiz Syed M. Abdullah[1], Mossaab Debbar[1], Nico Ueberschaar[2], Matthew T. Agler[1*]

[1]Plant Microbiosis Lab, Department of Microbiology, Friedrich Schiller University Jena, Germany
[2] Mass Spectrometry Platform, Friedrich Schiller University Jena, Germany

*** Corresponding author:**
Matthew T. Agler
Department of Microbiology, Plant Microbiosis Lab
Friedrich Schiller University Jena
Neugasse 23
07743 Jena, Germany
E-mail: matthew.agler@uni-jena.de
Tel: +49 (0)3641 9 49980

Contents:

Supplementary Figure 1 | Cross-feeding from leaf extract isolates
Supplementary Figure 2 | Growth curves *Pseudomonas* strains, phenotype on enriched R2A
Supplementary Figure 3 | Tagged *Pseudomonas* strains and CFU's
Supplementary Figure 4 | Raw measurements of biomass and mOrange2
Supplementary Figure 5 | Fluorescence and biomass measurements
Supplementary Figure 6 | Genetic similarity between three *Pseudomonas* strains
Supplementary Figure 7-12 | Metabolite uptake by *Pseudomonas* strains
Supplementary Figure 13 | Fluorescence and biomass measurements
Supplementary Figure 14 | CFU counts
Supplementary Figure 15 | *Pseudomonas* growth on *Ps* Fr+CA_2 spent media
Supplementary Figure 16 | *In-planta* inoculations and growth on *Pantoea* spent media
Supplementary Figure 17 | Inhibition test of *Pantoea* spent media and CAP on diverse *Pantoea* spent media
Supplementary Figure 18 | Metabolite uptake by *Ps* Fr-CA_5 from diverse *Pantoea* spent media
Supplementary Figure 19 | Fluorescence measurements

Supplementary Table 1 | Volume of bacterial suspensions in *Pseudomonas* co-cultures
Supplementary Table 5 | Average nucleotide identity between *Pseudomonas* strains
Supplementary Table 7 | Average nucleotide identity *Pantoea* strains.
Supplementary Table 9 | Oligonucleotides used in this study
Supplementary Table 10 | Composition of deuterated amino acid standard mix.
~~Supplementary Table 11 | Unique genome variants of each sequenced *Pseudomonas* strain~~

Supplementary Methods

Supplementary Notes

Extra References

Supplementary Figure 1

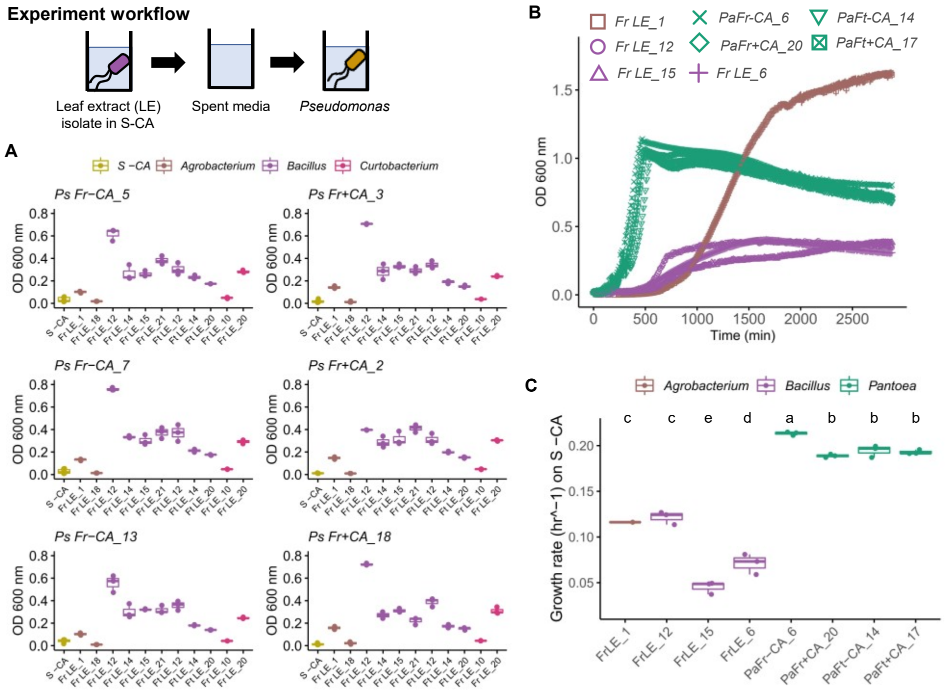

Supplementary Figure 1. Diverse isolates could cross-feed *Pseudomonas,* but they were probably outcompeted on sucrose by *Pantoea* (*Pa*). A) Growth of several *Pseudomonas* (Ps) isolates on spent media from different *Agrobacterium*, *Bacillus* and *Curtobacterium* isolates obtained from the leaf extracts (LE) of *F. robusta* and *F. trinervia*. B) Growth in S-CA medium of different *Agrobacterium* and *Bacillus* isolates versus the *Pantoea* isolates. C) Growth rates in S-CA medium of the same isolates as in panel B. Different letters indicate statistical differences after a Tukey test (*p* value <0.05).

Supplementary Figure 2

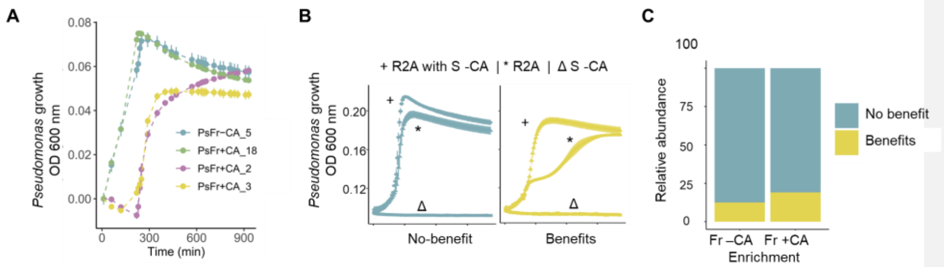

Supplementary Figure 2. The enrichments harbored metabolically diverse *Pseudomonas* isolates with different nutrient dependencies. A) Growth curve of two fast (*Ps* Fr-CA_5 and *Ps* Fr+CA_18) and two slow (*Ps* Fr+CA_2 and *Ps* Fr+CA_3) growing *Pseudomonas* isolates on *Pa* Fr$_{\text{-CA_6}}$ spent medium. B) Two different phenotypes observed across the *Pseudomonas* isolates when nutrients from the S-CA medium were added to their growth on R2A broth. C) Distribution of the two growth-phenotypes across the *Pseudomonas* isolates of each enrichment. In panels A and B each data point shows the average of three replicates and its corresponding standard error.

Supplementary Figure 3

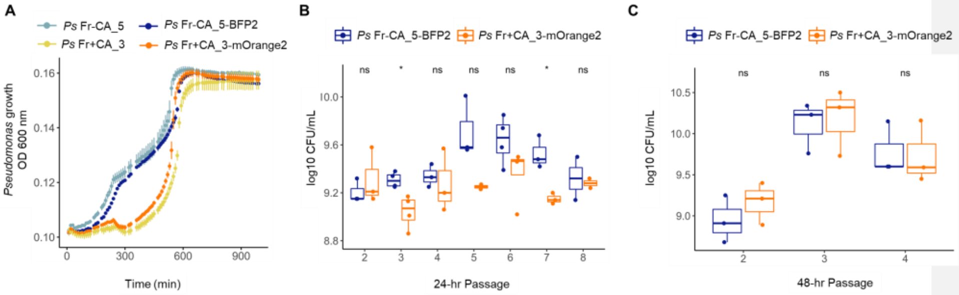

Supplementary Figure 3. Tagging did not affect the strains phenotypes. A) Growth of tagged versus non-tagged *Pseudomonas* (Ps) isolates on *Pantoea* (Pa) Fr-CA_6 spent media. Each data point is the average of three replicates and the corresponding standard error. ***Ps*****Fr-CA_5 and *Ps* Fr+CA_3 can persist together in ~~either~~ S-CA with *Pantoea* ~~or in S+CA medium~~.** B) CFU counts of each tagged *Pseudomonas* in the full community passaged every 24 hours C) CFU counts of each tagged *Pseudomonas* in the full community passaged every 48 hours. N=4 in panels B and C and D.

Supplementary Figure 4

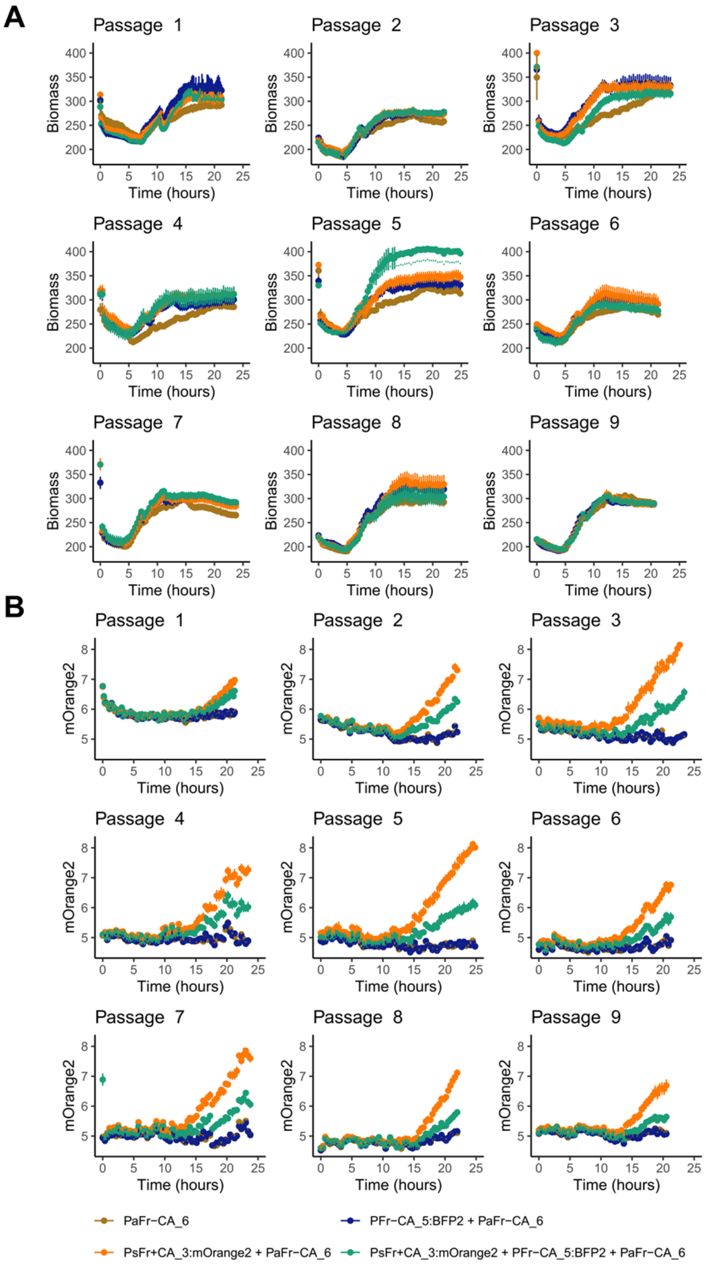

Supplementary Figure 4. A) Biomass signal in the co-cultures (each tagged *Pseudomonas* individually with *Pantoea*) and full community (both tagged *Pseudomonas* strains and *Pantoea* Fr-CA_6) throughout the passages. B) mOrange2 signal in the co-cultures and full community throughout the passages. Each data point is the average of four replicates with standard error.

Supplementary Figure 5

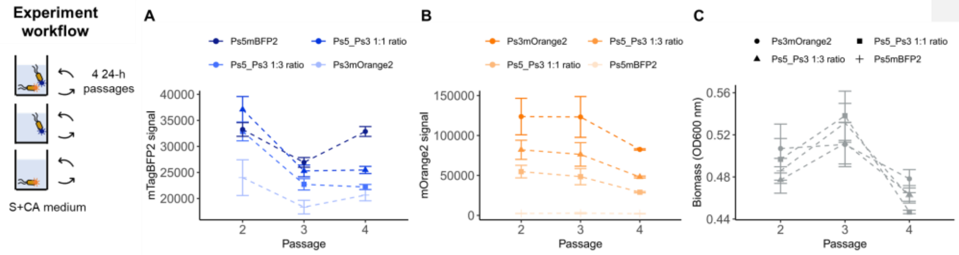

Supplementary Figure 5. Tagged *Pseudomonas* strains can coexist in S+CA medium. Fluorescence and biomass signals over four 24-h passages of the two tagged *Pseudomonas* growing on S+CA medium either in monoculture or together in two different ratios (1:1 or 1:3). In each plot data points show the average of three replicates and the corresponding standard error.

Supplementary Figure 6

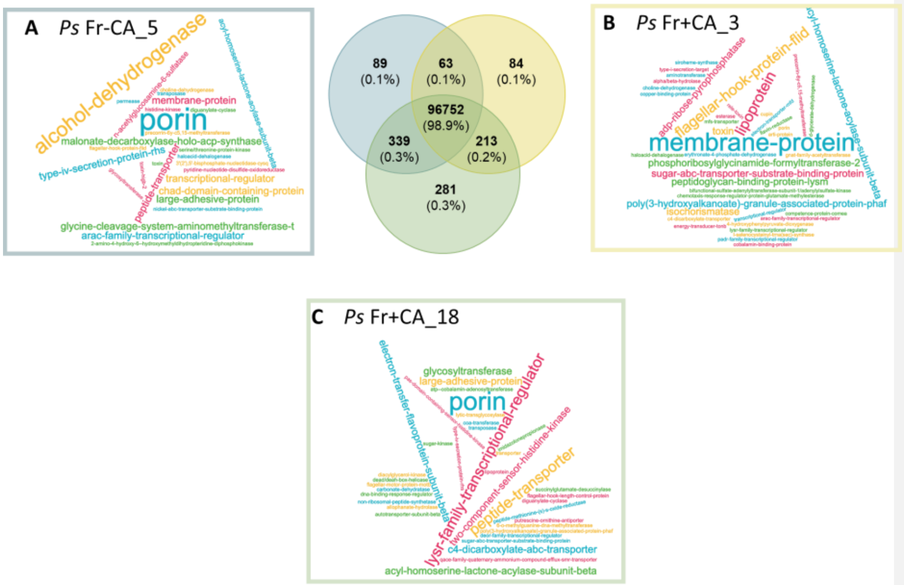

Supplementary Figure 6. *Pseudomonas* (*Ps*) isolates with distinct metabolic phenotypes have extremely similar genomes. The Venn Diagram in the center shows the number of single nucleotide variants across three *Pseudomonas* isolates after annotation with the reference genome of *P. siliginis* D26. Unique SNPs for each isolate with potentially disruptive mutations are shown for each *Pseudomonas* strain. The word size represents the relative frequency of disruptive SNPs in a particular gene product.

Supplementary Figure 7

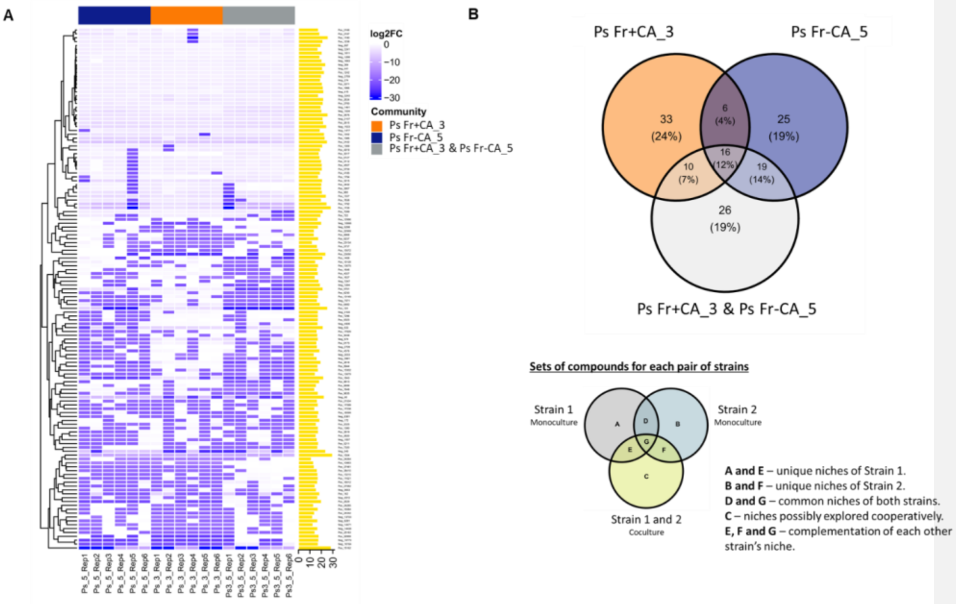

Supplementary Fig 7. Compounds taken up from the spent media of *Pantoea* Fr-CA_6 by either *P. siliginis* Fr+CA_3:mOrange2 and *Pseudomonas* Fr -CA_5:mTagBFP2 in monoculture, or both strains growing in co-culture. A) Heatmap of taken up compounds. Each column represents a different repetition (N=6). The strains and peaks have been clustered by Euclidean distance. The yellow bars on the right indicate the area (log10) of the peak in the spent media of *Pantoea* Fr-CA_6 before growth of the *Ps.* Each peak is identified with a unique number; for information of each one (ionization mode, retention time, m/z value and putative annotation) see Supplementary Table 6. B) Venn Diagram showing the sets of compounds taken up by each strain alone and when co-cultured. An explanation for easier interpretation of the different areas of the diagram is provided. In both panels A and B only metabolites with significant uptake are shown (log2 FC <-2, FDR<0.05).

Supplementary Figure 8

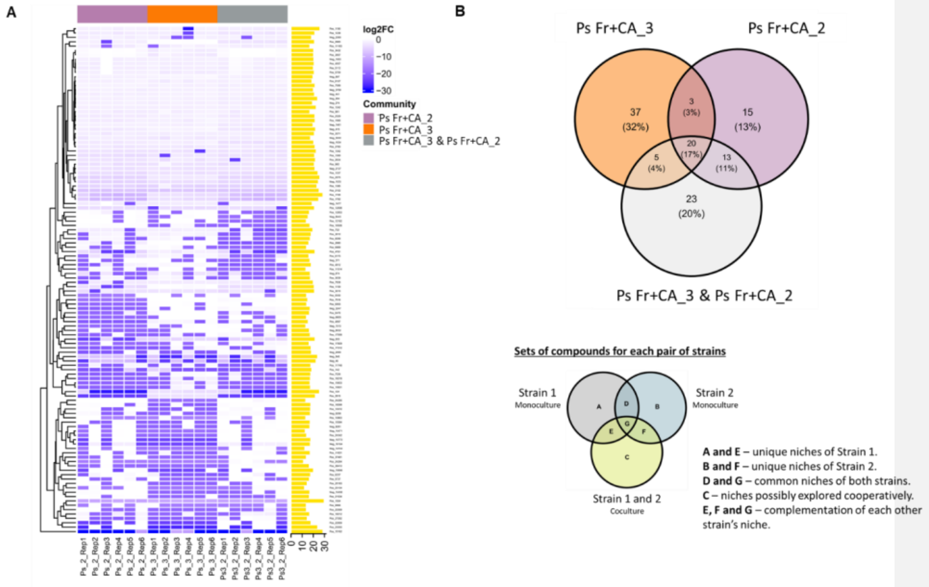

Supplementary Fig 8. Compounds taken up from the spent media of *Pantoea* Fr-CA_6 by either *P. siliginis* Fr+CA_3:mOrange2 and *Pseudomonas* Fr +CA_2 in monoculture, or both strains growing in co-culture. A) Heatmap of taken up compounds. Each column represents a different repetition (N=6). The strains and peaks have been clustered by Euclidean distance. The yellow bars on the right indicate the area (log10) of the peak in the spent media of *Pantoea* Fr-CA_6 before growth of the *Ps.* Each peak is identified with a unique number; for information of each one (ionization mode, retention time, m/z value and putative annotation) see Supplementary Table 6. B) Venn Diagram showing the sets of compounds taken up by each strain alone and when co-cultured. An explanation for easier interpretation of the different areas of the diagram is provided. In both panels A and B only metabolites with significant uptake are shown (log2 FC <-2, FDR<0.05).

Supplementary Figure 9

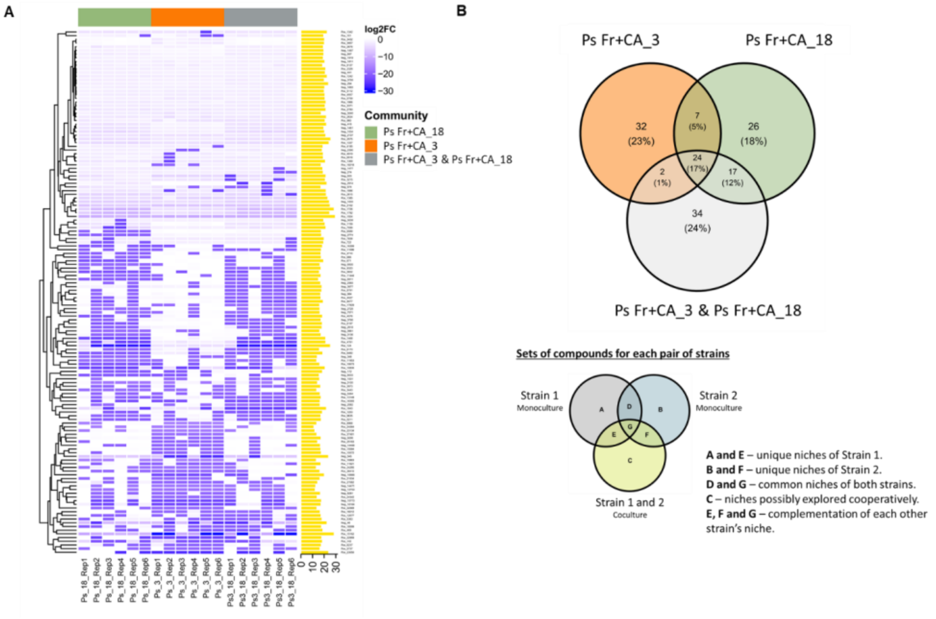

Supplementary Figure 9. Compounds taken up from the spent media of *Pantoea* Fr-CA_6 by either *P. siliginis* Fr+CA_3:mOrange2 and *P. siliginis* Fr +CA_18 in monoculture, or both strains growing in co-culture. A) Heatmap of taken up compounds. Each column represents a different repetition (N=6). The strains and peaks have been clustered by Euclidean distance. The yellow bars on the right indicate the area (log10) of the peak in the spent media of *Pantoea* Fr-CA_6 before growth of the *Ps.* Each peak is identified with a unique number; for information of each one (ionization mode, retention time, m/z value and putative annotation) see Supplementary Table 6. B) Venn Diagram showing the sets of compounds taken up by each strain alone and when co-cultured. An explanation for easier interpretation of the different areas of the diagram is provided. In both panels A and B only metabolites with significant uptake are shown (log2 FC <-2, FDR<0.05).

Supplementary Figure 10

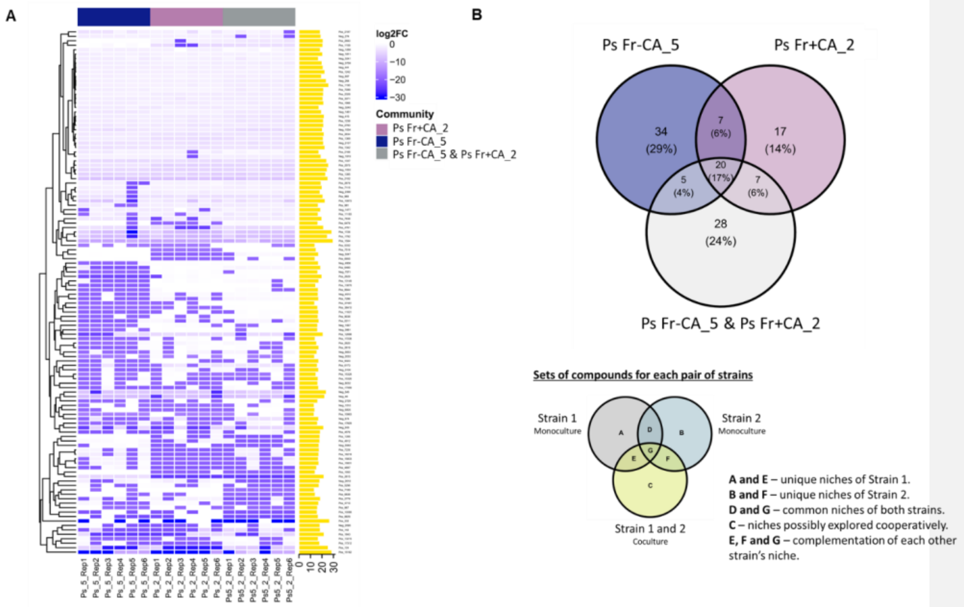

Supplementary Fig 10. Compounds taken up from the spent media of *Pantoea* Fr-CA_6 by either *P. siliginis* Fr-CA_5:mBFP2 and *P. siliginis* Fr +CA_2 in monoculture, or both strains growing in co-culture. A) Heatmap of taken up compounds. Each column represents a different repetition (N=6). The strains and peaks have been clustered by Euclidean distance. The yellow bars on the right indicate the area (log10) of the peak in the spent media of *Pantoea* Fr-CA_6 before growth of the *Ps*. Each peak is identified with a unique number; for information of each one (ionization mode, retention time, m/z value and putative annotation) see Supplementary Table 6. B) Venn Diagram showing the sets of compounds taken up by each strain alone and when co-cultured. An explanation for easier interpretation of the different areas of the diagram is provided. In both panels A and B only metabolites with significant uptake are shown (log2 FC <-2, FDR<0.05).

Supplementary Figure 11

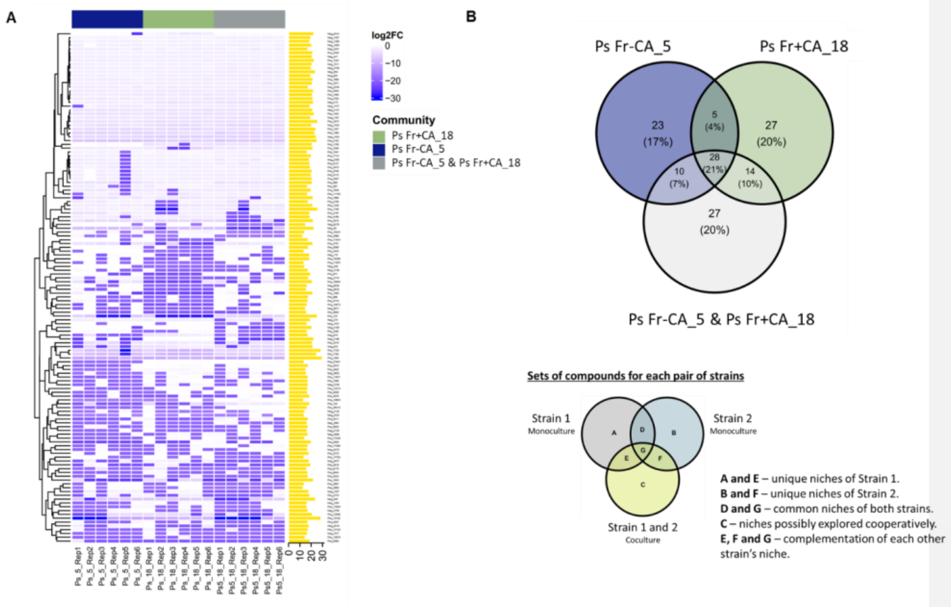

Supplementary Figure 11. Compounds taken up from the spent media of *Pantoea* Fr-CA_6 by either *P. siliginis* Fr-CA_5:mTagBFP2 and *P. siliginis* Fr +CA_18 in monoculture, or both strains growing in co-culture. A) Heatmap of taken up compounds. Each column represents a different repetition (N=6). The strains and peaks have been clustered by Euclidean distance. The yellow bars on the right indicate the area (log10) of the peak in the spent media of *Pantoea* Fr-CA_6 before growth of the *Ps.* Each peak is identified with a unique number; for information of each one (ionization mode, retention time, m/z value and putative annotation) see Supplementary Table 6. B) Venn Diagram showing the sets of compounds taken up by each strain alone and when co-cultured. An explanation for easier interpretation of the different areas of the diagram is provided. In both panels A and B only metabolites with significant uptake are shown (log2 FC <-2, FDR<0.05).

Supplementary Figure 12

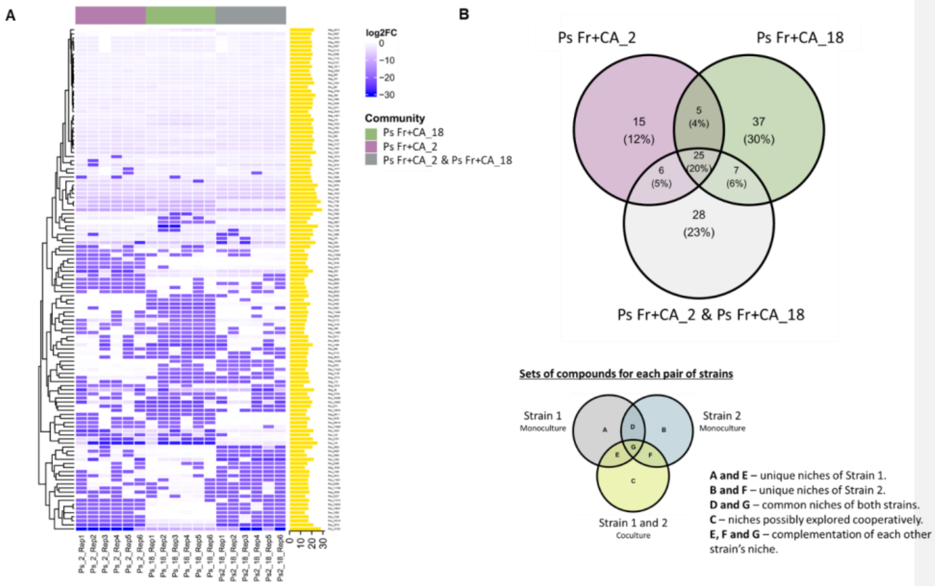

Supplementary Fig 12. Compounds taken up from the spent media of *Pantoea* Fr-CA_6 by either *P. siliginis* Fr+CA_2 and *P. siliginis* Fr +CA_18 in monoculture, or both strains growing in co-culture. A) Heatmap of taken up compounds. Each column represents a different repetition (N=6). The strains and peaks have been clustered by Euclidean distance. The yellow bars on the right indicate the area (log10) of the peak in the spent media of *Pantoea* Fr-CA_6 before growth of the *Ps*. Each peak is identified with a unique number; for information of each one (ionization mode, retention time, m/z value and putative annotation) see Supplementary Table 6. B) Venn Diagram showing the sets of compounds taken up by each strain alone and when co-cultured. An explanation for easier interpretation of the different areas of the diagram is provided. In both panels A and B only metabolites with significant uptake are shown (log2 FC <-2, FDR<0.05).

Supplementary Figure 13

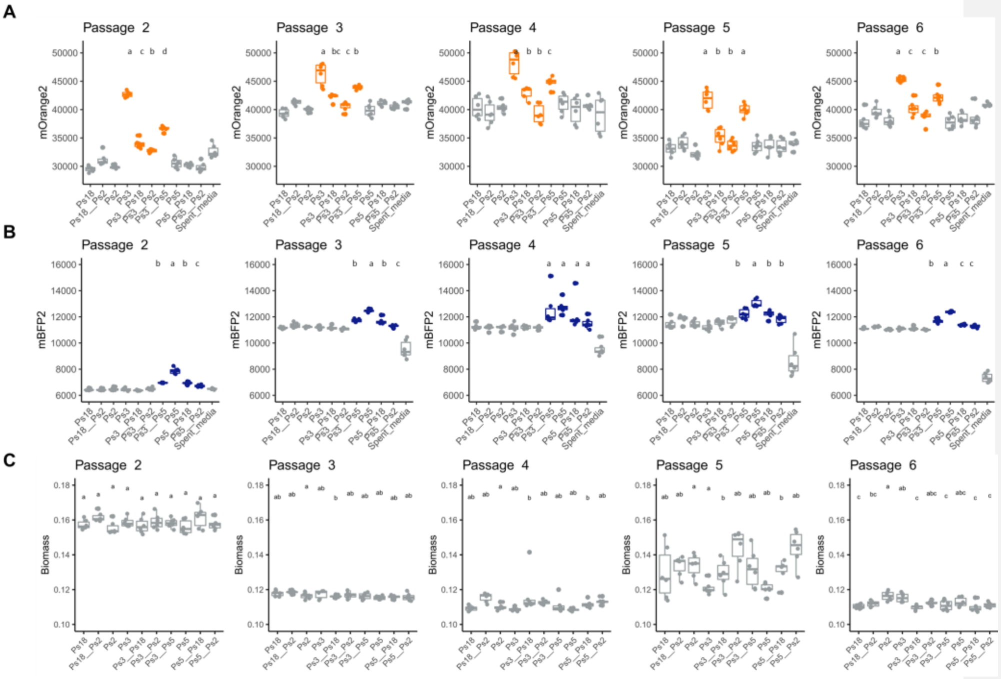

Supplementary Figure 13. Growth of tagged *Pseudomonas* (*Ps*) strains was reduced when co-cultured with the other strains, but overall biomass remained constant. Fluorescence and biomass measurements throughout the passages for each community of *Pseudomonas* (*Ps*). A) mOrange2 signal; only the communities including *Ps* Fr+CA_3:mOrange2 are colored in orange. B) mBFP2 signal; only the communities including *Ps* Fr-CA_5:mTagBFP2 are shown in blue. C) Biomass signal (OD 600 nm) for each community. In each plot different letters indicate significant differences after a Tukey test, N=6.

Supplementary Figure 14

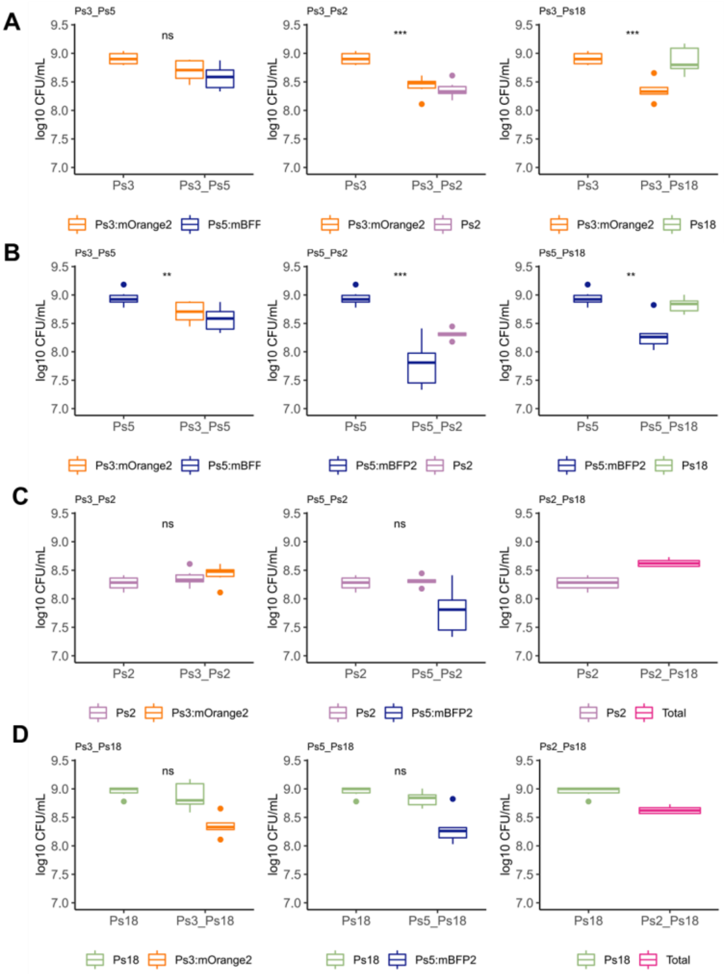

Supplementary Figure 14. ***Pseudomonas*** **strains with different growth patterns can coexist while cross-feeding from** ***Pantoea*** **Fr-CA_6 spent media.** Colony counts of four *Pseudomonas* strains growing either in monoculture or in pair with each of the other strains. Counts were taken after six passages on *Pantoea* 6 Fr-CA spent media. A) *P. siliginis* 3 Fr+CA:mOrange2 counts. B) *P. siliginis* 5 Fr-CA:mBFP2 counts. C) *Pseudomonas* 2 Fr+CA counts. D) *P. siliginis* 18 Fr+CA counts. In each case a t-test was performed between the counts in the monoculture and the counts in the co-culture (N=6, ns: $p > 0.05$, *: $p <= 0.05$, **: $p <= 0.01$, ***: $p <= 0.001$). *Pseudomonas* 2 and *P. siliginis* 18 colonies are not distinguishable, therefore only the total counts are reported and no significance test is shown.

Supplementary Fig 15

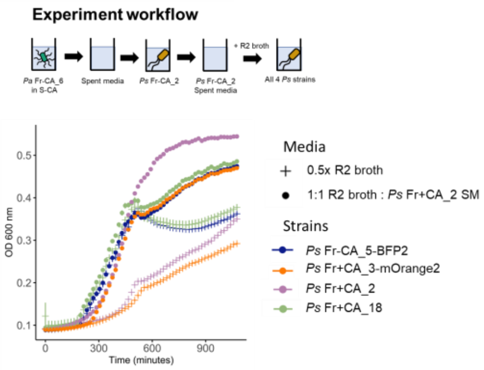

Supplementary Figure 15. Growth of *Pseudomonas* (*Ps*) strains is not inhibited by *Ps* Fr+CA_2. Growth of *Pseudomonas* (*Ps*) strains on the spent media of *Ps* Fr+CA_2, which was collected after growth of this strain on the spent media of *Pa* Fr-CA_6. Each data point shows the average of three replicates and its corresponding standard error.

Supplementary Figure 16

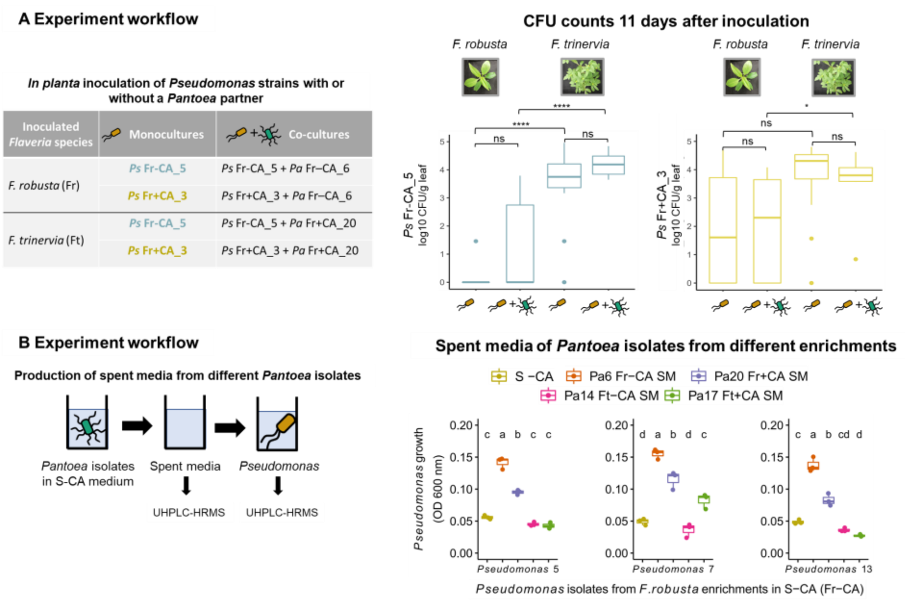

Supplementary Figure 16. Plant nutrient landscape might select on cross-feeding interactions. A) *In-planta* assay testing for the ability of two *Pseuduomonas* (Ps) strains to colonize leaves of *Flaveria robusta* and *F. trinervia* either when inoculated alone, or together with a *Pantoea* (*Pa*) isolate. The recovery of *Pseudomonas* isolates (*Ps* Fr-CA_5 or *Ps* Fr+CA_3) from *F. robusta* and *F. trinervia* leaves 11 days after inoculation is shown on the right panel. The experiments were performed twice fully independently (N= 8 leaves for *F. robusta,* N=12 leaves for *F. trinervia*). B) Production of spent media from four different *Pantoea* isolates. Their potential to support growth of three *Pseudomonas* isolates is shown on the right panel. Growth was recorded after 48 hours. Different letters indicate significant differences after a Tukey test (N=3).

Supplementary Figure 17

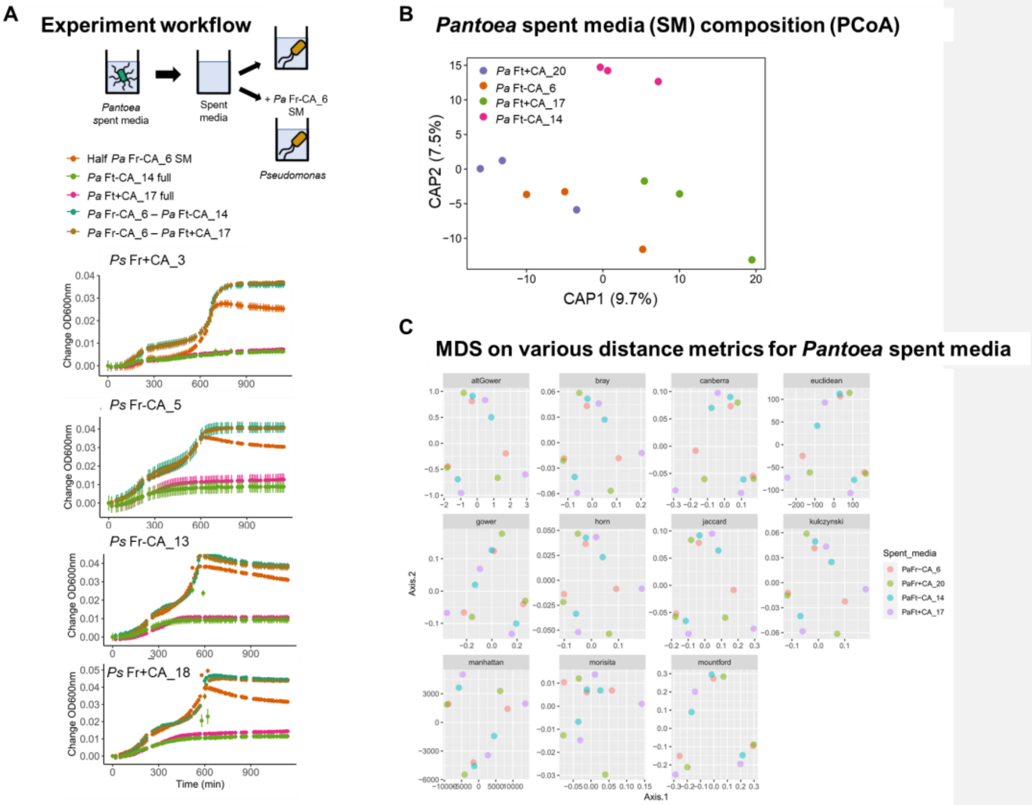

Supplementary Figure 17. **Low growth of *Pseudomonas* (*Ps*) isolates on spent media of *Pantoea* from *Flaveria trinervia* is not due to inhibitory metabolites or vast differences in composition.** A) Growth of several *Ps* isolates on *Pantoea* Ft-CA_14 and Ft+CA_17 spent media, either at full concentration, or in 1:2 combination with spent media of *Pantoea* Fr-CA_6. The curves are the average of three independent samples. The error bars at each time point show the standard error. B) Constrained analysis of principal components of all peaks (area>1.0E4) detected in the spent media of the different *Pantoea* isolates. A generalized logarithm transformation (Parsons et al, 2007) was applied to the data and the analysis was based on Euclidean distance matrix. (N=3). More details on the peaks detected in each spent media can be found in Supplementary Table 7. C) Different distance measurements tested on the same data set as in panel B with unconstrained principal coordinates analysis.

Supplementary Figure 18

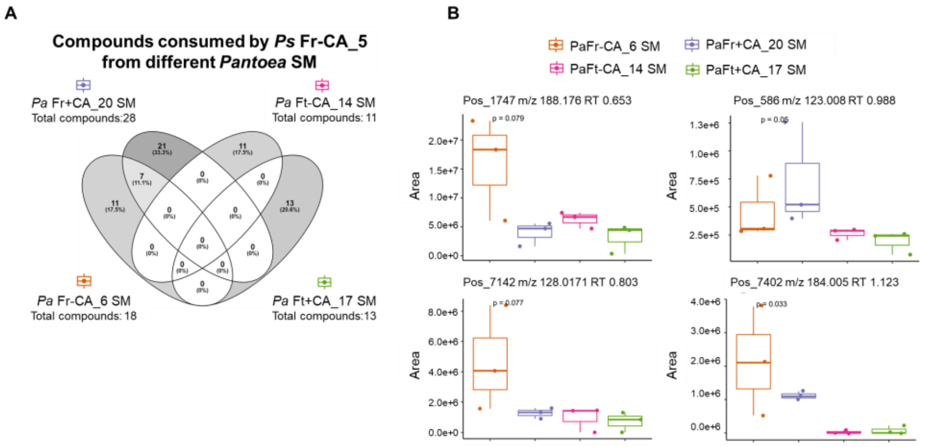

Supplementary Figure 18. Spent media from *Pantoea* (*Pa*) from the *Flaveria robusta* enrichments are seemingly better nutrient source for *Pseudomonas* (*Ps*) Fr-CA_5. A) Number of compounds significantly taken up (FDR<0.05, log2FC<-2) by *Pseudomonas* Fr-CA_5 from the different *Pantoea* spent media. B) Areas of compounds commonly taken up uniquely from the spent media of the two *F. robusta Pantoea* isolates. For each peak, the ionization mode in which it was detected (Pos=positive, Neg=negative), the m/z value and the retention time are shown. Each media had three replicates; *p* values are based on Kruskal-Wallis test.

Supplementary Figure 19

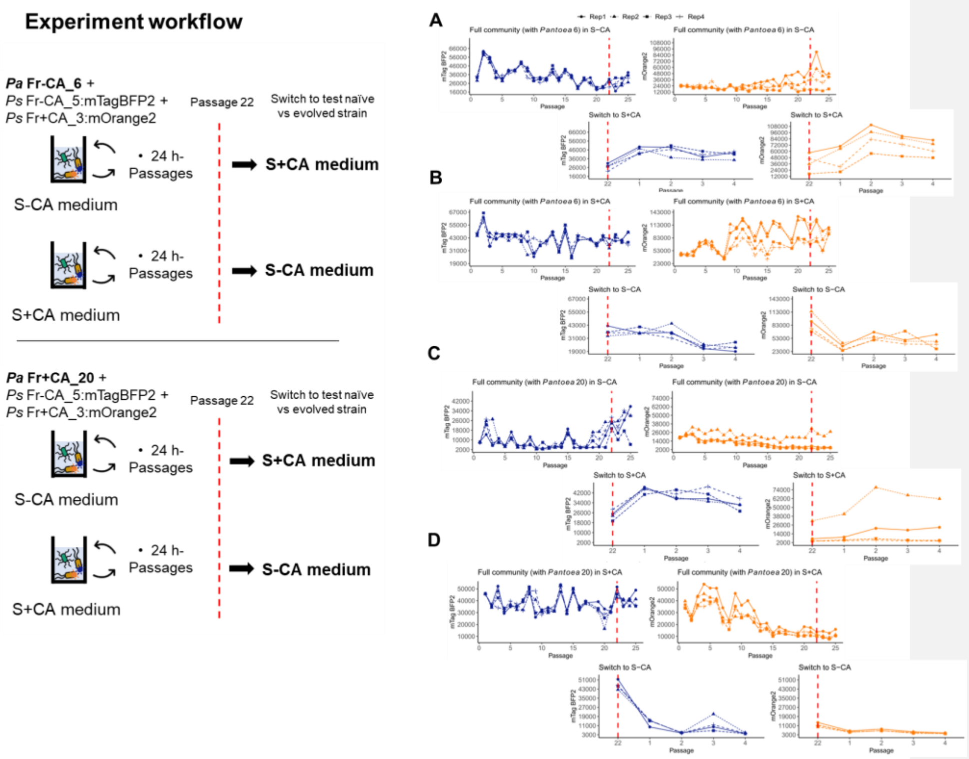

Supplementary Fig 19. Interactions between *Pantoea* and *Pseudomonas* followed distinct paths in each environment. Fluorescence signals (mTagBFP2 and mOrange2) in the full communities throughout the passages. Panels A and B show the communities with *Pantoea* Fr-CA_6 in S-CA and S+CA respectively. Panels C and D show the communities with *Pantoea* Fr+CA_20, also with S-CA and S+CA respectively. The red vertical line at passage 22 indicates the switch to the opposite medium. After the switch, the fluorescence was monitored for four more passages. Each condition had four replicates; all the raw values are shown in the figures.

Supplementary Table 1. Volume (µL) of bacterial suspension of *Pantoea* (Pa) and *Pseudomonas* (Ps) added as inoculum per well in each of the competition assays ~~(Experiments 4a, 4b and 4c)~~
.

Experiment	Community	***Pa* Fr-CA_6**	***Ps* FrCA_5: mBFP2**	***Ps* Fr+CA_3: mOrange2**	**Ps Fr+CA_2**	**Ps Fr+CA_18**	**1x PBS**
Growth of tagged *Ps* and *Pa* Fr-CA_6 in S-CA media over 8 and 4 passages (24 and 48h, respectively).	Pa Fr-CA_6 + Ps Fr-CA_5:BFP2	10	3.3	---	---	---	6.6
	Pa Fr-CA_6 + Ps Fr+CA_3:mOrange2	10	---	6.6	---	---	3.3
	Pa Fr-CA_6 + Ps Fr-CA_5:BFP2 + Ps Fr+CA_3:mOrange2	10	3.3	6.6	---	---	---
	Pa Fr-CA_6	10	---	---	---	---	9.9
Growth of tagged *Ps* in either monoculture or co-culture in S+CA over 4 24h-passages	Ps Fr-CA_5:BFP2 monoculture	---	10	0	---	---	10
	Ps Fr+CA_3:mOrange2 monoculture	---	0	10	---	---	10
	Ps Fr-CA_5:BFP2 + Ps Fr+CA_3:mOrange2 (1:1 ratio)	---	10	10	---	---	0
	Ps Fr-CA_5:BFP2 + Ps Fr+CA_3:mOrange2 (1:3 ratio)	---	5	15	---	---	0
Growth of *Ps* monocultures or in pairs on PaFr-CA_6 spent media over 6 24-h passages	Ps Fr+CA_3:mOrange2 monoculture	---	---	10	---	---	10
	Ps Fr-CA_5:BFP2 monoculture	---	10	---	---	---	10
	Ps Fr+CA_2 monoculture	---	---	---	10	---	10
	Ps Fr+CA_18 monoculture	---	---	---	---	10	10
	Ps Fr-CA_5:BFP2 + Ps Fr+CA_3:mOrange2	---	10	10	---	---	---
	Ps Fr+CA_2 + Ps Fr+CA_3:mOrange3	---	---	10	10	---	---
	Ps Fr+CA_18 + Ps Fr+CA_3:mOrange3	---	---	10	---	10	---
	Ps Fr+CA_2 + Ps Fr-CA_5:BFP2	---	10	---	10	---	---
	Ps Fr+CA_18 + Ps Fr-CA_5:BFP2	---	10	---	---	10	---
	Ps Fr+CA_2 + Ps Fr+CA_18	---	---	---	10	10	---

Supplementary Table 5.

Average nucleotide identity (%) between the genomes of several *Pseudomonas* (*Ps*) isolates.

	**Ps Fr-CA_5**	**Ps Fr+CA_3**	**Ps Fr+CA_18**
**Ps Fr-CA_5**		99.9909	99.9906
**Ps Fr+CA_3**	99.9909		99.9841
**Ps Fr+CA_18**	99.9906	99.9841	

Supplementary Table 7

Average nucleotide identity (%) between the genomes of several *Pantoea* (*Pa*) isolates.

	**Pa Ft-CA_14**	**Pa Fr+CA_20**	**Pa Ft+CA_17**	**Pa Fr-CA_6**
**Pa Ft-CA_14**		81.160	99.997	98.685
**Pa Fr+CA_20**	81.160		81.292	81.207
**Pa Ft+CA_17**	99.997	81.292		98.678
**Pa Fr-CA_6**	98.685	81.207	98.678	

Supplementary Table 9. Oligonucleotides used in this study.

Name	Target	Sequence 5' to 3'	References
B341F	16S rRNA V3/V4	CCTACGGGNGGCWGCAG	(1)/(2)
B806R	16S rRNA V3/V4	GGACTACHVGGGTWTCTAAT	
8F	16S rRNA	AGAGTTTGATCCTGGCTCAG	
1492R	16S rRNA	GGTTACCTTGTTACGACTT	
FWD Tn*5/7*_gt	Veryfing the Tn7 insertion	ATGGTGAGCAAGGGCGAG	(3)
REV_Tn*5/7*_gt	Veryfing the Tn7 insertion	CAACAGGAGTCCAAGCTCAG	
Tn*7*_gt1	Genotyping the Tn7 backbone	GAATTACAACAGTACTGCGATGAG	
Tn*7*_gt2	Genotyping the Tn7 backbone	GATCAACTCTATTTCTCGCGGG	
Tn*7*_gt3	Genotyping the Tn7 backbone	TACATAACGGACTAAGAAAAACACTACAC	
FWD_uidA	*uidA* gene (*E. coli*)	AACAGGTGGTTGCAACTGGA	
REV_uidA	*uidA* gene (*E. coli*)	TTGCTGAGTTTCCCCGTTGA	

Supplementary Table 10. Concentration of deuterated amino acids in CELL FREE AMINO ACID MIX (U-D, 98%), from Cambridge Isotope Laboratories, Inc. (MA, USA). Lot# PR-29964

Deuterated amino acid	Weight percentage (m/m)
Alanine_D	6.80%
Arginine_D	3.10%
Asparagine_D	4.40%
Aspartate_D	4.90%
Glutamine_D	4.50%
Glutamic Acid_D	10.30%
Histidine_D	1.20%
Lysine_D	8.70%
Leucine_D|Isoleucine_D	18.90%
Methionine_D	1.90%
Phenylalanine_D	6.30%
Proline_D	2.60%
Serine_D	3.80%
Threonine_D	5.10%
Tryptophan_D	2.60%
Tyrosine_D	3.00%
Valine_D	6.50%

Supplementary Methods

Recovery and metabolomic analysis of leaf apoplast fluid from lab plants

Cuttings from *Flaveria robusta* and *F. trinervia* were grown in pots containing a mixture of soil (66%) perlite (33%) and Substral Osmocote® NPK (Mg) 17-9-11 (2) (4 g/L of soil mixture). The plants were kept at an average day/night temperature of 25 °C/22 °C and a photoperiod of 16 hours in our lab. The leaf apoplast of *F. robusta*, *F. trinervia* and *F. linearis* was characterized by UHPLC-HRMS. Samples of the three species were collected over the course of two years: *F. linearis* was sampled on two occasions four months apart (March 2020 and July 2020); each time five plants were grown and ten samples were collected (2 samples per plant). For *F. robusta*, three different sets of cuttings were sampled: one on March 2020 (4 different plants, sampled twice each), June 2020 (two different plants sampled twice) and April 2021 (2 different plants, sampled four and two times). For *F. trinervia*, four plants were sampled in March 2020, along with the other two species (2 samples per plant).
To obtain the apoplast samples, well developed leaves were sampled and placed in a 60-cc syringe, which was filled with sodium phosphate (100 mM, pH 6.5). The plunger was pushed until the 50-cc mark to eject air, then pulled until the 55-cc mark and released back to the 50-cc mark; this was repeated several times until the leaves lost buoyancy. To recover the apoplast fluid wash (AFW), the leaves were placed on a sheet of parafilm, rolled around a 15-mL tube and placed inside a 50 mL tube. After three minutes of centrifugation at 2500 x g, the recovered AFW was transferred to a clean 1.5 mL tube. After storage at -20 °C, the samples were spiked with an internal standard of deuterated amino acids and subjected to metabolomic profiling via untargeted UHPLC-HRMS (Supp Table 10).

In vitro enrichment and characterization of leaf microbiomes under different nutrient regimes

Plant sampling and preparation of leaf extracts

Cuttings from *F. robusta* and *F. trinervia* were grown in pots as described earlier and kept in our lab at an average day/night temperature of 25 °C/22 °C and a photoperiod of 16 hours. After roots were developed (~3 weeks), they were transplanted into an outdoor garden (Jena, Germany) to allow natural colonization by microorganisms. After two months, well-developed leaves from both species were sampled, weighed and washed three times in sterile water to remove dirt and insects. Leaf extracts were prepared by macerating the washed leaves with a sterile pistil, adding 1 mL of 1X PBS with 0.02% Silwet and vortexing for 10 seconds, followed by quick centrifugation to precipitate down the larger segments. The supernatant was transferred to a clean tube, mixed with glycerol to a final 20% v/v concentration and stored at -80 °C. The number of live bacterial cells in the glycerol stocks was estimated by plating serial dilutions on Reasoner's 2A agar (R2A) and calculating CFU/mL.

Enrichment of leaf microbiomes from Flaveria trinervia *and* Flaveria robusta

The leaf extracts were enriched *in vitro* over 12 passages on two different sucrose-based minimal media. First, a preculture was generated by inoculating ~1000 cells in 15 mL of basic M9 broth (2 mM MgSO4*7H20, 0.2 mM CaCl2, 1 µg/mL biotin, 1 µg/mL thiamine, 11 mM sucrose and trace elements: 134 µM EDTA, 31 µM $FeCl_3$-6H_2O, 6.2 µM $ZnCl_2$, 0.76 µM $CuCl_2$-2H2O, 0.42 µM $CoCl_2$-2H2O, 1.62

µM H_3BO_3, 0.081 µM $MnCl_2$-$4H_2O$), supplemented with 0.2% w/v casamino acids (Difco) 200 mM NH_4Cl and 200 µg/mL of cycloheximide to limit eukaryotic growth. The cultures were incubated at 26 °C and 220 rpm for 72 hours. One fraction of the culture was used to prepare 20% glycerol stocks. The remaining volume was centrifuged (5000 x g for 5 min), the pellet washed twice in 1X PBS and resuspended to a final OD_{600nm} of 0.3 in fresh 1x PBS. Five microliters of the suspension were inoculated in a 2 mL 96 well plate containing 1 mL M9 media supplemented with NH_4Cl (33 mM) and either no casamino acids (S-CA) or 0.2% m/v casamino acids (S+CA). Each enrichment condition was run in triplicates. The plate was incubated at 26 °C and 220 rpm. Every 48 hours, the cultures were homogenized by pipetting up and down and 5 µL of each well were transferred to a new plate with fresh media. The OD_{600nm} was measured at each passage and the procedure was repeated 12 times. In the first two passages, cycloheximide was added in the same concentration as before. At the last passage, 700 µL of each well were pelleted for DNA extraction by centrifuging at 20000 x g for 10 min. The remaining volume of the three replicates were combined to prepare 20% glycerol stocks, which were stored at −80 °C.

Characterization of culture-independent bacterial diversity in enrichments

The bacterial communities in the twelfth passage were characterized by 16S rRNA gene amplicon sequencing. Cell pellets were resuspended in 600 µL SDS extraction buffer (SDS extraction buffer, 10% filter sterilized SDS, 100 mM Tris pH 8.0, 200 mM NaCl, 2 mM EDTA) and transferred to 2 mL screw-cap tubes containing ~0.2 g of glass beads (0.25-0.50 mm, ROTH). The mixture was incubated at 37°C for 10 min, followed by bead beating for 30 sec at 1400 rpm in a BioSpec Mini-Beadbeater-96 and 4 minutes centrifugation at 13400 rpm. The supernatant was transferred into new 1.5 mL sterile Eppendorf ~~tubesand~~tubes and 200 µL of 5 M potassium acetate were added to precipitate the SDS. After 5 minutes of centrifugation at 13400 rpm, the supernatant was discarded and the pellet was resuspended in 1x TE buffer. ~~recovered in a clean tube.~~ The DNA was purified using 1.5x volume of Sera-Mag™ magnetic carboxylate modified particles and eluted in 50 µL of 10 mM TrisHCl (pH 8.0). The purified DNA was stored at −20 °C until further amplification. Amplification of the 16S V3-V4 region was carried out in a two-step PCR; on the first step, the samples were amplified using the bacterial primers B341F and B806R(1,2) (Supp Table 9). The PCR mastermix contained: 8 µL KAPA 5x GC buffer, 0.3 mM KAPA DNTPs, 0.8 µL Kapa HiFi polymerase (KAPA Biosystems), 0.075 µM of each primer, 1 µL of template and 28.4 µL NFW. The amplification settings were 2 min denaturing at 95 °C, followed by 15 cycles of denaturation at 98 °C for 30 seconds, annealing at 50 °C for 30 sand elongation at 72 ° C for 40 sec, followed by a final extension at 72 °C for 2 minutes. The PCR products were enzymatically cleaned with Antarctic phosphatase and Exonuclease I (New England Biolabs, Inc) (0.5 µL each enzyme with 1.22 µL Antarctic phosphatase buffer at 37 °C for 30 minutes followed by 80 °C for 15 min). In the second step, concatenated primers were used; these were designed to include an Illumina adapter P5 (forward) or P7 (reverse), an index sequence, a linker regionand the B341F and B806R primers. Each sample was amplified in triplicates; the Mastermix consisted of 3 µL KAPA 5x GC buffer, 0.3 µM KAPA DNTPs, 0.3 µL KAPA HiFi polymerase (KAPA Biosystems), 0.015 µM of each primer, 1 µL of the cleaned product from the first PCR and 8.25 µL of

Formatted: Highlight

NFW. The cycling settings were: 2 min denaturing at 95 °C, followed by 25 cycles of denaturation at 98 °C for 30 seconds, annealing at 50 °C for 30 sand elongation at 72 ° C for 40 sec, followed by a final extension at 72 °C for 2 minutes. Samples that did not amplify with these settings were amplified with 30 cycles instead. Triplicate reactions were pooled and purified with 0.65x volume magnetic beads and eluted in 10 mM TrisHCl and run in a 1.5% agarose gel. The brightness of each band in the gel was measured in ImageJ (version 1.52a) and used to adjust the volume of each sample in the final library. After combining all samples, the pool was purified once again with 0.8x volume Sera-Mag beads and quantified in a Qubit (Thermo Fisher Scientific, Inc). The library was denatured and then loaded onto a MiSeq lane spiked with 10% PhiX genomic DNA to ensure high sequence diversity and sequenced in 500 cycles (2x250 bp).

Amplicon sequencing data was split on the indices and the adapters were trimmed from the read ends using Cutadapt. The data was clustered into amplicon sequencing variants "ASVs" using the R package dada2 (version 1.18.0: (4), setting the quality filter at (truncLen=c(200,200)). Next, the sequences were dereplicated to remove redundant reads, followed by denoising using the error rate and calling of ASVs in the forward and reverse reads. When merging the forward and reverse reads, only overlapping regions were kept. Chimeric sequences were removed and obtained a sequence table with the merged data. For taxonomy assignment, the Silva database (v132) was used. Ordination analyses were carried out in R (version 4.0.4, (5)) with the packages *phyloseq* (version 1.34.0; (6)) and *vegan* (version 2.5-7; (7)).

Characterization of culturable bacterial diversity in original leaf extracts and in enrichments

Isolates were recovered from the initial leaf extract glycerol stocks and from the glycerol stocks from the twelfth enrichment passage of each condition. Serial dilutions from the glycerol stocks were prepared in 1x PBS and spread in R2A plates. From each condition, 25 random isolates were selected and re streaked to recover pure cultures (150 isolates total). To minimize isolate adaptation to plating, no further cultivation was performed and pure isolates were grown in R2A broth for 48 hours to prepare 20% glycerol stocks. All isolates were identified via DNA extraction and Sanger sequencing of the 16S rRNA gene. Liquid cultures in R2A broth of each isolate were pelleted by centrifugation at 20000 x g for 10 min and stored at -20 °C. DNA extraction and purification was done as described in the previous section. Whole 16S rRNA gene was amplified with the universal primers 8F and 1492R (Supp Table 9). The PCR mastermix contained: 8 µL KAPA 5x GC buffer, 0.32 µM KAPA DNTPs, 0.8 µL KAPA HiFi polymerase (KAPA Biosystems), 0.27 µM of each primer, 1 µL template and 24.2 µL nuclease free water (NFW). The amplification consisted of 2 min denaturing at 95 °C, followed by 30 cycles of denaturation at 95 °C for 30 seconds, annealing at 50 °C for 30 sand elongation at 72 ° C for 1:30 min, followed by a final extension at 72 °C for 5 minutes. and the products were Sanger sequenced (Eurofins Genomics, Germany). The sequences were trimmed in R (version 4.0.4) using the package *sangeranalyseR* (version 1.26.0; (8)) with default parameters. The resulting fasta file was "BLASTed" against the NCBI 16S rRNA gene database. The first ten hits were used to identify taxonomy at the genus level using a least common ancestor algorithm in MEGAN6 with percent to cover set to 70 and the minimum percent identity to 97 (Huson et al., 2016).

Whole genome sequencing

Whole genome sequencing was carried on three *Pseudomonas* sp. (*Ps* Fr-CA_5, *Ps* Fr+CA_3 and *Ps* Fr+CA_18) and four *Pantoea* sp. isolates (*Pa*Fr-CA_6, *Pa*Fr+CA_20, *Pa*Ft-CA_14 and *Pa*Fr+CA_17). For this, a modified DNA extraction protocol was used. The isolates were grown overnight in 2 mL of R2A broth at 28 °C and 220 rpm. The cultures were pelleted by centrifugation (20000 xg for 5 min), resuspended in 600 µL SDS extraction buffer and transferred to 2 mL screw-cap tubes containing ~0.2 g of glass beads (0.25-0.50 mm, ROTH). The mixture was incubated at 37°C for 10 min, followed by bead beating for 30 sec at 1400 rpm in a BioSpec Mini-Beadbeater-96 and five minutes centrifugation at 20000 x g. The supernatant was transferred into new 1.5 mL sterile Eppendorf tubes to which 100 µg/mL of Proteinase K (Sigma) were added. The mixture was incubated for one hour at 37 °C, followed by 10 min at 80 °C to deactivate the enzyme. Once the tube had cooled, 10 µg/mL RNAse A was added and incubation was carried at 37 °C for 30 min. To remove proteins, an equal volume of phenol: chloroform: isoamyl alcohol (25:24:1) were added to each sample, followed by 10 min centrifugation at 4 °C and 20000 x g. The top layer was transferred to a clean tube where an equal volume of chloroform / isoamyl alcohol (24:1) was added. The centrifugation step was repeated and again the top layer was transferred to a new tube. One-tenth volume of 3M sodium acetate and 2.5 volumes of 100% ethanol were added and the tubes were left overnight at -20 °C. To pellet the DNA, the tubes were spun for 40 minutes at 20000 x g and 4 °C; the supernatant was removed and the pellet was washed with 200 µL of 70% ethanol and left to air dry. Finally, the DNA was resuspended in 100 µL of 10 mM TrisHCl. The samples were sent to Microbial Genome Sequencing Center (Pittsburgh, USA) for sequencing on the NextSeq 2000 (Illumina) platform at a depth of 300 MBp (~50x for *Pseudomonas* strains and ~60x for *Pantoea* strains).

Whole genome assembly, annotation, ANI calculation and single nucleotide polymorphism detection

For each isolate, the raw forward and reverse reads were trimmed using Trimmomatic (version 0.39), which removed the Illumina adaptersand trimmed at quality below 15 in a 4-base wide sliding window. The assembly of the genomes was done using SPADES (3.14.1) with the default parameters in "isolate" mode. Average nucleotide identity between the different isolates of each genera was calculated in Kbase (9). First, the final scaffolds from the SPADES assembly were imported into Kbase the average nucleotide identity was calculated using the FastANI app.

Sequence variants (SNPs and indels) compared to the *Pseudomonas siliginis* D26 reference genome (*Pseduomonas* Genome Database (10), assembly accession GCF_001605965.1) were then called. To do so, we used SNIPPY (version 4.6.0) with default parameters in "Multi" mode to map the raw sequencing reads from each of the three *Pseudomonas* strains. This resulted in one variant table for each strain including the chromosome location, the gene it was in (if any) and the predicted effect. An R script was then used to parse these tables to determine which variants were shared or unique for each *P. siliginis* isolate. Unique variants were extracted for each isolate and were manually filtered for potentially disruptive mutations (disruptive inframe insertions/deletions, frameshift variants, missense variants, stop gains and initiator codon variants). The resulting lists are provided in Supplementary

Table 11. These lists were used to make word clouds (http://www.wordclouds.com) where the word size represents the relative frequency of disruptive variants in a particular gene product. All scripts and step-by-step instructions are available on Figshare.

Assessment of the isolates carbon preferences and cross-feeding potential

Evaluation of the isolates carbon preference

To test metabolic dependencies among the communities, we grew all 150 isolates individually in S-CA and S+CA media. Single colonies taken from R2A plates were inoculated in triplicates in a 96-well flat bottom plate, containing 200 µL of media per well. The plates were incubated at 26 °C and 220 rpm in a shaker for 24 h. The OD_{600nm} was measured right after inoculation and after 24 h. A second passage over the same media was done to verify the observed growth was not due to carryover of R2A nutrients. An isolate was categorized as growing if its final OD_{600nm} was ≥0.1. Of the isolates that showed growth without CA supplementation, we chose several and measured their growth rate in the S-CA medium. For this, the isolates were pre grown in S-CA broth for 24 hours at 220 rpm and 28 °C. The cells were washed twice with 1X PBS, centrifuging each time at 5000 rpm for 5 min and discarding the supernatant in between. The washed cells were resuspended in 1X PBS and diluted to an OD_{600nm} of 0.2. In a 96-well plate, 20 µL of the bacterial suspension were mixed with 180 µL of S-CA broth. All strains were inoculated in triplicates. The plate was incubated in a VersaMax Tunable Microplate Reader (Molecular Devices, CA) for 48 hours at 28 °C, taking OD_{600nm} measurements every 10 min and mixing in between reads. To determine the growth rate of each curve, we calculated the first derivative in 30-min windows across the length of the curve, then we selected the periods where the derivative was larger than the average variation (i.e., the exponential phase) and calculated the mean derivative for those time points.

Several isolates that could not grow without CA were tested for amino acid auxotrophy on a modified version of the S-CA media, where sucrose was replaced by 22 mM glucose. Additionally, we checked whether they could consume sucrose in presence of other nutrients. For this, they were precultured in R2A broth overnight at 28 °C and 220 rpm, harvested and diluted to an OD_{600nm} of 0.2 as detailed above and inoculated in a 96-well plate containing 180 µL of each of the following S-CA: R2A broth: sterile water ratios; 1:0:0.8, 1:0.2:0.6, 0:0.2:1.6. Each strain was inoculated in triplicates in each of the five conditions by adding 20 µL of the cell suspension (final OD_{600nm} 0.02). The plate was incubated in the plate reader with the same settings as before.

Production of spent media and evaluation of cross-feeding interactions

To generate spent media Pa Fr-CA_6 was grown in 250 mL flasks with 80 mL of S-CA (sucrose-only) media at 26 °C and 220 rpm. After 48 hours, the cells were separated from the spent media by centrifuging at 5000 x g for 5 minutes and filter sterilizing twice through a 0.22 µm PES filter. Spent media were subjected to untargeted metabolomics (see details in the Metabolomics section) or used further to assay growth of *Pseudomonas*. The *Pseudomonas* isolates were grown in R2A broth for 24 hours at 28 °C and 220 rpm; the cells were washed twice and resuspended in 1x PBS to an OD_{600nm} of 0.2. In 96-well plates, 180 µL of the sterile spent media and 20 µL of the bacterial suspension were

mixed (final OD_{600nm} 0.02). As a negative control, the isolates were also inoculated in full strength S-CA media. After or during 48 hours of incubation at 28 °C and 220 rpm, growth was recorded (OD_{600nm}). To measure taken up compounds, the plate was centrifuged at 5000 x g for 5 min and the supernatants were transferred to a clean plate and stored at -20 °C until injection in the UHPLC-MS. The same procedure was followed to test the cross-feeding potential of several *Agrobacterium*, *Bacillus* and *Curtobacterium* isolates from the leaf extracts of *F. robusta* and *F. trinervia* (FrLE and FtLE). Spent media from these isolates was produced in S-CA medium and tested as growth medium for several *Pseudomonas* strains. The metabolite uptake from FrLE_12 and FrLE_1 was also determined by UHPLC-HRMS.

Competition between *Pseudomonas* strains while cross-feeding

Tagging of Pseudomonas *strains with fluorescent proteins and competition assay*

To test whether faster growing *Pseudomonas* isolates would outcompete slow growing isolates either when growing along with *Pantoea* or in its spent media, we tagged the *Pseudomonas* Fr-CA_5 and Fr+CA_3 with the fluorophores mTagBFP2 and mOrange2 respectively, using the delivery plasmid systems pMRE-Tn7-140 and pMRE-Tn7-144 developed by Schlechter et al. (3). In brief, *E. coli* ST18 containing each plasmid was grown overnight in LB broth + 5-aminolevulinic acid (ALA) (50 µg/mL) + Amp (100 µg/mL) + Gen (15 µg/mL) + Cam (15 µg/mL) at 30°C and 200 rpm. The *Pseudomonas* strains were grown in LB without ALA or antibiotics under the same incubation settings. On the following day, 25 mL of LB + ALA + antibiotics were inoculated with 500 µL of the overnight *E. coli* cultures and 25 mL of LB without antibiotics were inoculated with 1000 µL of the *Pseudomonas* culture and grown until an OD_{600nm} of about 0.7. The cells were harvested by mild centrifugation (2000 x g for 5 min)and after discarding the supernatant, they were resuspended in 1X PBS by gently inverting the tubes. The *Pseudomonas* and the corresponding *E. coli* cultures were mixed in three different ratios; 1:3, 1:1 and 3:1 considering the OD_{600nm} of the suspensions. The combined cells were harvested as before, resuspended in 100 µL of 1X PBS and drop spotted in an LB + ALA plate containing 100 µL 1 M CaCl2 to increase conjugation efficiency. The plates were incubated at 30 °C overnight; the next day a colony was suspended in 1 mL 1X PBS and different volumes (100-300 µL) were spread in LB plates containing antibiotics and incubated at 30 °C for several days. Colonies that appeared in the plates were checked for fluorescence under UV light (330 nm) and in a Axiozoom Stereomicroscope (Zeiss). To rule out the selected colonies were not *E. coli*, a PCR using the specific primers FWD_uidA and REV_uidA (Supp Table 9) was performed. Additionally, the presence of the fluorescent protein coding sequence was checked with the primers FWD_Tn5/7_gt and REV_Tn5/7_g (Supp Table 9). In both cases, the Mastermix consisted of: 1x Buffer B (Biodeal), 0.4 mM dNTPs (Carl Roth), 0.2 µM of each primer, 2.5 mM $MgCl_2$ and 0.25 µL Taq DNA polymerase (Biodeal). For the *E. coli* specific primers, the cycling conditions were: 3 min at 95 °C, 30 cycles of 30 sec at 95°C, 30 sec at 57.5 °C and 1 min at 72 °C, followed by a 5 min final extension at 72 °C. For the set of primers targeting the transposon, the cycling was the same, except for the annealing temperature, which was set at 56.5 °C. Once the colonies were confirmed to be fluorescent *Pseudomonas*, they were grown in liquid LB + Gen + Cam at 37 °C and 200 rpm to cure them from the plasmid. The efficacy of this last

step was confirmed via PCR with the primers FWD_Tn7_gt, Rev_Tn7_gt and Tn7_gt (Supp Table 9) targeting the plasmid backbone. The Mastermix was prepared as detailed before, except the concentration of each primer was 0.12 µM and the annealing temperature was set to 62 °C. To determine whether producing the fluorescent protein had a fitness cost on the strains, their growth on PaFr-CA_6 spent media was assessed. Precultures of the tagged and non-tagged strains were prepared in R2A broth as usual, inoculated to a final OD_{600nm} of 0.02 in 180 µL of spent media and incubated for 24 hours at 220 rpm and 28 °C. To check if either tagged strain had a fitness advantage over the other one, they were combined in different ratios considering their OD_{600nm} (1:1, 3:1 and 1:3) and grown in R2A broth under the same conditions as before. CFU's were counted after 24 h under UV light to differentiate each strain.

Competition experiments using tagged Pseudomonas *strains*

Using the tagged strains, three competition assays were established (see Supp Table 1 for details on the inoculation volumes of each strain). 1) The tagged strains were combined individually with *Pantoea* Fr-CA_6 or together in a full mix (both *Pseudomonas* strains and *Pantoea*) and inoculated in black 96-well plates containing 180 µL of S-CA media. The plate was covered with an AeraSeal (Excel Scientific, Inc.) sealing film and incubated in a a BioLector I (m2p-labs Beasweiler, Germany) at 500 rpm, 30 °C with humidity control. Every 24 hours, 5 µL of each well were transferred to 195 µL of fresh media. This was repeated for a total of eight passages. The full community was also subcultured every 48 hours for four passages. Each community had four replicates. After each round, the plate was opened under sterile conditionsand 2 µL were sampled for serial dilutions and CFU counts, whereby *Pseudomonas* Fr-CA_5 and *Pseudomonas* Fr+CA_3 were distinguishable due to fluorescence. During the runs, growth of *Pseudomonas* Fr+CA_3 was monitored by normalizing the red filter channel signal (gain=100) to the biomass signal (gain= 35) and this ratio was used to compare its growth in co-culture with *Pantoea* alone vs. together with *Pseudomonas* Fr-CA_5. 2) The tagged strains were passaged in S+CA in either monoculture or two co-culture ratios (1:1 and 1:3, being the latter higher on PsFr+CA_3:mOrange2) every 24 hours for a total of four passages. Each community had four replicates. 3) Both tagged strains and two additional *Pseudomonas* isolates: PsFr+CA_2 and PsFr+CA_18, were grown in either monoculture or in each possible pair on the spent media of PaFr-CA_6 for six 24-h passages. Samples for metabolite uptake were collected after the first passage following the same procedure described above. CFUs of each community were counted in LB plates after the last passage. Colonies were checked for fluorescence under UV light (330 nm) and in a Axiozoom Stereomicroscope (Zeiss). In the Ps Fr+CA_2 and Ps Fr+CA_18 combination, only total number of cells were recorded as it is not possible to distinguish their morphologies.

In all three experiments, the isolates were precultured in R2A broth for 24 hours and washed and resuspended in 1x PBS as detailed earlier. ~~Experiments 4b and 4c~~The second and third experiments were carried out in 96-well plates (Greiner 655101) containing 180 µL of the corresponding media and 20 µL of inoculum. The plates were incubated at 28 °C and 220 rpm in an orbital shaker and every 24 hours, 5 µL of each well were passaged to a new plate with 195 µL of fresh spent media. Additionally, the OD_{600nm} and the fluorescence of the mTagBFP2 and mOrange2 were read at the time of

passaging in a Tecan Plate Reader (Infinite 200Pro), using the following parameters for each channel: (blue signal: Ex/Em WL: 402/457 nm, gain = 78, 3 flashes, Z-position: 18249 µm, 2x2 reads per well with a border of 1250 µm; red signal: Ex/Em WL: 550/580 nm, gain = 134, 10 flashes, Z-position: 19190 µm, 2x2 reads per well with a border of 1400 µm; biomass signal: absorbance at 600 nm, 10 flashes, 1 reading per well).

Evaluating relation of cross-feeding efficiency and colonization to host species.

In-planta testing of *Pseudomonas* colonization

The *Pseudomonas* strains Fr-CA_5 and Fr+CA_3and the *Pantoea* strains Fr-CA_6 and Fr-CA_14 were grown in LB agar plates. The colonies were resuspended in 10 mM MgCl2 with 0.02% silwet and diluted to an OD_{600nm} of 0.002. *Flaveria robusta* and *F. trinervia* cuttings were prepared in the lab under controlled conditions as described ~~in Exp. 1~~before. Three-week old plants were used for testing the bacterial inoculation. *F. robusta* cuttings were inoculated with each *Pseudomonas* alone or in combination with *Pa* Fr-CA_6. *F. trinervia* cuttings were inoculated in the same way, except using *Pa*Fr-CA_14 for the co-inoculations. In the co-inoculations, the bacterial suspensions contained each strain at an OD_{600nm} of 0.002.

Two fully independent experiments were performed for each species; for *F. robusta*, a total of 8 leaves were infiltrated and for *F. trinervia*, 12 leaves. The inoculation was carried out on the underside of the leaves, using a needless syringe. After inoculation, the plants were arranged in trays (each treatment in a separate tray to avoid cross-contamination), covered with a plastic lid to maintain humidity and placed inside a growth chamber with a photoperiod of 16 hours and a day/night temperature of 22/18 °C. After 10 days, the leaves which had been inoculated were harvested, surface sterilized first with 2% bleach + 0.02% Triton, followed by 70% ethanol and then washed three times with sterile water. Next, the leaves were thoroughly crushed and suspended in 10 mM MgCl2. Serial dilutions were prepared and plated in LB agar. The plates were incubated for 48 h after which the colonies were counted.

Cross-feeding potential of diverse Pantoea isolates towards Pseudomonas

Pantoea isolates from other enrichments (Fr +CA, Ft -CA and Ft +CA) were tested for their cross-feeding potential towards *Pseudomonas*. The same procedure described ~~for Exp. 3 was followed~~above to obtain the spent media and prepare the *Pseudomonas* precultures was followed. The assays were carried out in triplicates in 96-well plates by inoculating 180 µL of spent media with 20 µL of the corresponding bacterial suspension. The plates were incubated at 28 °C and 220 rpm in an orbital shaker. Growth (OD_{600nm}) was determined after 48 hours. Samples for metabolomics were taken at the end of the experiment as described earlier.

To test for inhibitory effects, the spent media of *Pantoea* isolates *Pa* Ft-CA_14 and *Pa* Ft+CA_17 were diluted 1:2 with spent media *of Pa* Fr-CA_6 and inoculated as before. Growth (OD_{600nm}) of the *Pseudomonas* strains was monitored over the course of 24 hours.

Metabolomics

Ultra-high performance liquid chromatography - high resolution mass spectrometry

Both the apoplast fluid wash from leaves (AFW) and the samples from the cross-feeding experiments were analyzed by untargeted metabolomics. Before injection, the samples were mixed with a standard mix of deuterated amino acids (U-D 98% Cell Free Amino Acid Mix from Cambridge Isotope Laboratories, Inc.) to a final concentration of 20 µg mix/mL sample (individual concentrations of each amino acid are shown in Supp Table 10). Ultra-high performance liquid chromatography coupled with high resolution mass spectrometry was carried out using a THERMO (Bremen, Germany) UltiMate HPG-3400 RS binary pump, WPS-3000 auto sampler which was set to 10 °C and which was equipped with a 25 µL injection syringe and a 100 µL sample loop. The column was kept at 25 °C within the column compartment TCC-3200. Chromatography column was used THERMO Accucore® C-18 RP (100 × 2.1 mm; 2.6 µm) using the following gradient of Eluent A (water with 2% acetonitrile and 0.1% formic acid) and Eluent B (pure acetonitrile), respectively at a constant flow rate of 0.4 mL/min: 0 min (100% and 0%), 0.2 min (100% and 0%), 8 min (0% and 100%), 11 min (0% and 100%), 11.1 min (100% and 0%), 12 min (100% and 0%). Mass spectrums were recorded with THERMO QExactive plus orbitrap mass spectrometer coupled to a heated electrospray source (HESI). Column flow was switched at 0.5 min from waste to the MS and at 11.5 min again back to the waste, to prevent source contamination. For monitoring two full scan modes were selected with the following parameters. Polarity: positive; scan range: 80 to 1200 m/z; resolution: 70,000; AGC target: 3 × 106; maximum IT: 200 ms. General settings: sheath gas flow rate: 60; auxiliary gas flow rate 20; sweep gas flow rate: 5; spray voltage: 3.0 kV; capillary temperature: 360 °C; S-lens RF level: 50; auxiliary gas heater temperature: 400 °C; acquisition time frame: 0.5 - 11.5 min. For negative mode, all values were kept instead of the spray voltage which was set to 3.3 kV.

Metabolomics data analysis

The raw data files were converted into mzML format in MSConvert (ProteoWizard, Version 3.0.19246-075ea16f5). To reduce the size of the files, we filtered them by peak picking with the Vendor algorithm, followed by threshold peak filtering using absolute intensity at 1.0E2. Next, we imported them into MzMine (version 2.40.1) for processing. The spectrums were first separated into positive and negative ionization modes. The baseline was corrected at 1.0 m/z bin width and using the asymmetric correction method. The peaks were then selected by centroid detection and by setting the noise level at 1.0E2. The chromatograms were built with the ADAP Chromatogram builder. The settings to build the chromatograms were slightly modified for each run by verifying the right integration of the deuterated amino acids peaks. The minimum group size in a number of scans was set to 2-3, the group intensity threshold was either 1.0E4 or 6.0E4, the minimum highest intensity was set at the same value as the group intensity and the m/z tolerance was set at 0.001 m/z. The generated chromatograms were deconvoluted with the Wavelets (ADAP) algorithm, setting a signal to noise threshold of 10.0, a minimum feature height of 1.0E4, a coefficient/area threshold of 50, peak duration range 0-1 and a RT wavelet range of 0-0.1. Furthermore, the peaks were aligned together using the Join Aligner with m/z tolerance of 0.001, retention time tolerance of 0.1 min and setting the

weight for m/z and RT at 75 ppm and 25 ppm respectively. Finally, the matrix of negative and positive mode peaks, with their corresponding ID, m/z, retention time and area were exported into R (version 3.6.0). Using an in-house script, uncommon (in less than 50% of the samples in the run) and small peaks (area <104) were filtered out. In the case of the AFW samples, the areas were corrected by the infiltration ratio.

To get a first overview of the distribution of the peaks across plant species, we conducted a principal coordinate analysis, constrained by plant species using Euclidean distance on the corrected peak matrix with the R packages phyloseq and vegan. Before plotting the data, the areas under the curve of each peak were transformed by glog2(x)= log2*{[x+sqrt(x2+a2)]/2}, where “a” is a constant with a default value of 1. We used this transformation as it has been shown to emphasize biological variation over technical variation in metabolomic data (Parsons et al., 2007). To compare the amino acids, the peak matrix was first annotated against a custom library of 570 compounds including all proteinogenic amino acids (MSMLS, Sigma Aldrich) which had been developed in the same equipment and UHPLC-MS method we used. This was carried out in R with an in-house script (See data availability) by setting the m/z tolerance at 0.002 and the RT tolerance at 0.2 min. The area of each amino acid was corrected towards its deuterated internal standard as follows:

$$\text{Corrected area} = \text{Area of amino acid in sample} * \frac{\bar{x}\ \text{area of deuterated amino acid in all samples in the run}}{\text{area of deuterated amino acid in sample}}$$

Next, we calculated the concentration of each amino acid, based on the known concentrations of the standard (Supp Table 10).

To find out which compounds had been taken up via cross-feeding, the spent media was analyzed before and after growth of the consumer isolates. To focus exclusively on uptake of large peaks, only peaks present in high amounts in the spent media (before growth of the consumers) were considered. This cutoff was defined for each dataset and ranged between 1.0E4 and 1.0E5. The log2 fold change, p-value and FDR were calculated for each peak. Metabolites with a fold change lower than -2 (after growth / before growth) and an FDR <0.05 were defined as significantly taken up. Venn Diagrams were created using the online tool Venny (version 2.1.0) and heatmaps were built in R with the package ComplexHeatmaps (11). The resulting peak tables were annotated against the custom library as described above.

Supplementary Notes

Analysis of reasons for differences in Pantoea cross-feeding

Pantoea did not inhibit *Pseudomonas* growth (Supp Fig 17) and metabolome analysis suggested only minor differences in their exudates (see CAP analysis ~~in~~ and principal coordinates in Supp Fig 17 *p* value=0.7713) with several compounds previously identified as taken up (hypoxanthine and spermidine) present in all (Supp Table 8). We did identify a few compounds that were taken up uniquely from either of the *Pantoea* Fr spent media that were not abundant in the other *Pantoea* strains (Supp Fig 18 and Supp Table 8). These are good metabolite candidates for what drove *Pseudomonas* growth, although we could not yet annotate them.

Detailed analysis of experimental evolution of cross-feeding

In the S-CA communities with *Pantoea* Fr-CA_6 (Supp Fig 19a), *P. siliginis* Fr-CA:5mBFP2 maintained a stable growth throughout the passages and the continuous passaging did not lead to major changes in the community, as the final intensity growth in S+CA ($\bar{x}$=43393) resembled that of the first passage of the community exposed to S+CA (Fig 19b, $\bar{x}$=46279, *p* value=0.0877). Growth of *P. siliginis* Fr+CA_3:mOrange2 increased in three of the replicates over time (Fig 19a). Evolving to be better in S-CA positively affected growth in +CA growth, leading to higher intensities at the 4th passage after the switch ($\bar{x}$=66032) versus in the community exposed to S+CA (Fig 19b, $\bar{x}$=44939, *p* value=0.0312). In the S+CA communities with *Pantoea* Fr-CA_6, *P. siliginis* Fr-CA:5mBFP2 was stable throughout the passages but decreased when switched to a S-CA (Fig 19b), reaching generally lower values ($\bar{x}$=22958) than those measured in the original S-CA communities (Fig 19a, $\bar{x}$=32097, *p* value=0.0044). On the other hand, *P. siliginis* Fr+CA_3:mOrange2 apparently evolved a better response to a lack of amino acids, as the final intensity after the switch to S-CA (Fig 19b, $\bar{x}$=43964) was slightly higher than that in the original S-CA community, although this difference was not significant (Fig 19a, $\bar{x}$=24007, *p* value=0.069).

In the co-cultures with *Pantoea* Fr+CA_20 in absence of CA, *P. siliginis* Fr-CA:5mBFP2 experienced an increase towards the end of the experiment, suggesting a development of better cross-feeding interactions (Fig 19c). However, the passaging did not improve the overall cross-feeding interaction with this *Pantoea* isolate, as the intensity reached after the switch ($\bar{x}$=32388) was lower than the intensity in the beginning of the S+CA community (Fig 19d, $\bar{x}$=45663, *p* value=0.0007). The intensity of *P. siliginis* Fr+CA_3:mOrange2 in general decreased over time when together with *Pantoea* Fr+CA_20and the addition of CA did not have an effect of half of the replicates. However, in replicate 2 changes must have occurred because when CA were added, the intensity increased to quite higher levels (61806) than those in the first passage of the S+CA community (Fig 19d $\bar{x}$=36152). Co-culturing with *Pantoea* Fr+CA_20 in the S+CA communities seemed to have a negative effect on the interactions with both *P. siliginis* Fr-CA_5:mBFP2 and *P. siliginis* Fr+CA_3:mOrange2 as evidenced by the low values achieved after the switch to S-CA (Fig 19d, $\bar{x}$=4194 and $\bar{x}$=4196 respectively)and much lower than those in the S-CA communities (Fig 19c $\bar{x}$=8111 and $\bar{x}$=18620, p<0.0001).

Other references cited here:

1. Muyzer G, de Waal EC, Uitterlinden AG. Profiling of complex microbial populations by denaturing gradient gel electrophoresis analysis of polymerase chain reaction-amplified genes coding for 16S rRNA. Appl Environ Microbiol. 1993;59(3):695–700.

2. Caporaso JG, Kuczynski J, Stombaugh J, Bittinger K, Bushman FD, Costello EK, et al. QIIME allows analysis of high-throughput community sequencing data. Nat Methods. 2010;7(5):335–6.

3. Schlechter RO, Jun H, Bernach M, Oso S, Boyd E, Muñoz-Lintz DA, et al. Chromatic bacteria – A broad host-range plasmid and chromosomal insertion toolbox for fluorescent protein expression in bacteria. Front Microbiol. 2018;9:3052.

4. Callahan B, McMurdie PJ, Rosen M, Han A, Johnson A, Holmes S. DADA2: High-resolution sample inference from Illumina amplicon data. Nat Methods. 2016;13(7):581–3.

5. R Core Team. R: A language and environment for statistical computing [Internet]. Vienna, Austria: R Foundation for Statistical Computing; 2020. Available from: https://www.R-project.org/

6. McMurdie PJ, Holmes S. phyloseq: An R package for reproducible interactive analysis and graphics of microbiome census data. PLOS ONE. 2013;8(4):61217.

7. Oksanen J, Blanchet GF, Friendly M, Kindt R, Legendre P, McGlinn D, et al. vegan: Community Ecology Package [Internet]. 2020. Available from: https://CRAN.R-project.org/package=vegan

8. Chao KH, Barton K, Palmer S, Lanfear R. sangeranalyseR: Simple and Interactive Processing of Sanger Sequencing Data in R. Genome Biol Evol. 2021;13(3):evab028.

9. Arkin AP, Cottingham RW, Henry CS, Harris NL, Stevens RL, Maslov S, et al. KBase: The United States Department of Energy Systems Biology Knowledgebase. Nat Biotechnol. 2018;36(7):566–9.

10. Winsor GL, Griffiths EJ, Lo R, Dhillon BK, Shay JA, Brinkman FSL. Enhanced annotations and features for comparing thousands of *Pseudomonas* genomes in the *Pseudomonas* genome database. Nucleic Acids Res. 2016;44(D1):D646-653.

11. Gu Z, Eils R, Schlesner M. Complex heatmaps reveal patterns and correlations in multidimensional genomic data. Bioinformatics. 2016;32(18):2847–9.
